# Human intergroup coordination in a hierarchical multi-agent sensorimotor task arises from concurrent co-optimization

**DOI:** 10.1038/s41598-025-97574-3

**Published:** 2025-04-28

**Authors:** Gerrit Schmid, Daniel A. Braun

**Affiliations:** Faculty of Engineering, Computer Science and Psychology, Institute of Neural Information Processing, Ulm University, 89081 Ulm, Germany

**Keywords:** Motor control, Sensorimotor processing, Decision

## Abstract

Division of labor and specialization are common principles observed across all levels of biological organisms and societies, including humans that often rely on specialized roles to achieve a shared goal in complex coordination tasks. Understanding these principles in a quantitative fashion remains a challenge. In this study, we explore a novel experimental paradigm where two specialized groups of human players—a sensor group and an actor group—collaborate to accomplish a shared sensorimotor task of steering a cursor into a target. With all decision-makers initially unaware of their contribution and in the absence of verbal communication, the study explores how the group dynamics evolve over time, evaluating performance in terms of learning speed, group coherence and intergroup coordination. To gain quantitative insights, we simulate different computational models, including Bayesian learning and bounded rationality models, to describe human participants’ behavior. We also relate our findings to perceptual control theory, which emphasizes hierarchical control systems in which information flows bidirectionally between levels. Our results show that both human participants and model-based simulations (Bayesian and bounded rational agents) successfully complete the task. Over time, mutual information between actors and sensors increases, and cooperative behavior emerges within the groups. Interestingly, model-free hierarchical reinforcement learning fails to account for the observed data, being overwhelmed by task variability. In contrast, model-based approaches can be shown to generalize to larger groups and more complex network structures in evolutionary simulations. Our findings highlight the importance of internal models and concurrent co-optimization in facilitating adaptive coordination, offering insights into distributed information processing mechanisms.

## Introduction

In complex systems, modularity and specialization play a pivotal role, essentially ensuring robustness and efficiency of a compound system based on division of labor.^1^ This is particularly evident in biological systems, where hierarchical structures govern the organization and function of organisms at various scales. For instance, consider the human brain, where billions of specialized neurons work together to process information and coordinate responses. Crucially, each neuron does not need to understand the entire system, it only needs to perform its specific role. Similarly, each level of the hierarchy handles a specific level of complexity, allowing the system as a whole to function efficiently without any single part being overwhelmed. Coordination is also prevalent in human societies as well as social insects and other animal groups, where multiple specialized agents have to work together to achieve common goals.^2–6^ Multi-agent systems with artificial intelligence^7–10^ seek to emulate the same principles for engineered systems. Most often, multi-agent cooperation arises from the necessity for interaction of simple decision-makers that do not have the ability to tackle complex problems alone,^11^ be it because of limited information processing capabilities,^12,13^ restricted access to sensory input,^14,15^ a narrow action space,^16^ or the need to aggregate diverse preferences.^17^

In this study we are particularly interested in division of labour and specialization in sensorimotor tasks involving prelinguistic coordination amongst groups of humans. While specialization in human group decision-making has been previously investigated involving verbal feedback or information exchange,^18,19^ specialization in shared sensorimotor tasks has received much less attention. In the psychological literature, sensorimotor coordination among humans is often studied as joint action,^20,21^ where actors share goals and intentions. One mechanism that enhances such coordination is entrainment, where individuals synchronize their behaviors, such as matching rhythms or movements, to facilitate collaboration.^22–25^ This synchronization can be observed in various settings, from musicians playing in an orchestra to teammates working on a project.^26^ Movement synchrony is crucial in these interactions, as it fosters a sense of connection and cooperation between individuals. Moreover, when individuals move in synchrony, they are more likely to perceive their group as cohesive, which is known as perceived entitativity, further enhancing collaboration and mutual understanding.^27^ Such synchrony can even be shown to be underpinned by shared neural mechanisms, enabling individuals to anticipate and adapt to each other’s actions in real-time, creating a seamless flow of coordinated activity.^28^ Another mechanism facilitating joint action is leader-follower dynamics, where designated leaders guide the process by setting goals, making decisions, and providing direction, while followers adapt their actions accordingly.^29,30^ This dynamic can enhance the efficiency and effectiveness of collaborative tasks by providing clear structure and reducing ambiguity, offering valuable insights into how hierarchical structures can emerge naturally in group settings and contribute to the overall success of joint actions.

Quantitative models of how agents coordinate by sharing tasks or communicate high-level information, often assume that agents have access to each other’s actions, intentions, or information about the task. In particular, models of decentralized decision-making, such as those based on multi-agent reinforcement learning, have shown how agents can effectively coordinate by exchanging intentions or communicating directly.^31,32^ These frameworks emphasize how coordination can be achieved by distributing tasks across agents, with each agent contributing specialized information or control to the larger system. Similarly, decentralized and hierarchical decision-making frameworks have been used to model scenarios where agents rely on high-level plans to coordinate their actions and share sub-tasks under uncertainty.^33^ These models assume that agents, while working independently, can communicate and align their strategies, facilitating the coordination of complex tasks. Finally, a number of studies have examined dyadic sensorimotor interactions in game-theoretic settings by translating payoff matrices in classical $2\times 2$-matrix games into haptic coupling forces.^34–38^

While most of the above studies have focused on sensorimotor coordination between individuals, human group and intergroup sensorimotor coordination has been investigated more rarely, for example in the context of musical ensembles^39^ or in the context of social loafing in group sports like tug-of-war.^40^ Previously, we have investigated human coordination in a multi-agent sensorimotor task with neuron-like decision-making, dubbed the Neuron Game,^16^ where each neuron-like decision unit is impersonated by a human player. In the Neuron Game, groups of humans solve a sensorimotor task together, where each individual contributes a different skill to steer a cursor from a starting to a target position. However, all individuals operate in the same group of abstraction processing both sensory and motor information.

To investigate specialization and intergroup coordination, in our current study we separate individuals into two different groups with sensory and motor specialization, respectively. Each individual again contributes a distinct skill, but unlike the previous experiments, only a distinct subgroup of players (the sensor group) are able to observe the environment, while only another subgroup of players (the motor group) can act upon the environment. The goal of the game is that both groups learn to work together to solve a sensorimotor task, making them a system consisting of sensors and actors coupled by intergroup coordination. Within each subgroup, all players receive the same input, have to learn about their unique skills and also have to coordinate their skills. By dividing human participants into two distinct specialized groups, we effectively transform the original Neuron Game’s task into a complex of nested tasks, where each layer consists of a modified version of the original Neuron Game experiment. This nested control structure is reminiscent of perceptual control theory (PCT) that posits that behavior is the control of nested loops driven by perceptual perturbations.^41–45^ In this framework, an organism’s actions are guided by a hierarchy of (potentially nested) control systems, each with a specific goal, typically called reference value. These systems work together to maintain the perception of the environment at the desired state. Higher-level systems in the hierarchy set the goals for lower-level systems, allowing for complex and specialized behaviors.^46^ Individuals maintain stability in their actions through hierarchical feedback processes, where the coordination of actions is informed by continuous adjustment to disturbances.^45,47,48^

Importantly, no individual in such hierarchical control loops has oversight over the entire process, but only controls their local environment. This is not dissimilar from the scenario encountered by a neuron within a brain that is unaware of the consequences of its own actions on the environment or other regions of the brain. From a computational perspective we can ask whether there exist analogous principles that govern the collaboration among humans and the coordination of cells within a brain. In this analogy the groups of human individuals in our experiment ressemble a miniature artificial ”brain” made up of low-capacity decision-making units that together solve problems of sensorimotor coordination. In this multi-agent setting, each agent can be thought of as solving a specialized control problem for which the actions of others introduce disturbances. For a more quantitative understanding, we study different multi-agent learning models that can solve the credit assignment problem and emulate the observed intergroup coordination. In particular, we investigate Bayesian learning with internal models, i.e. statistical inference to integrate noisy sensory information to guide the decision-making process.

In the following we start out with the Results section including a more detailed description of the experiment, measures to quantify learning and cooperation within each subgroup as well as between the subgroups. Additionally, we provide two models: one to simulate the human participants as Bayesian learners, a method often used to describe human sensorimotor learners,^49^ and one to describe them as bounded rational decision-makers, where motor control is decision making^50^ of many units. Furthermore, we investigate in simulations how one could further evolve the sensor-actor-systems to contain more layers that contribute to the performance. In our Discussion we relate our findings to the existing literature before we summarize the main part of the paper in the Conclusion. In the last section, Methods, we detail the methodological approach used in the paper.

## Results

**Fig. 1:**
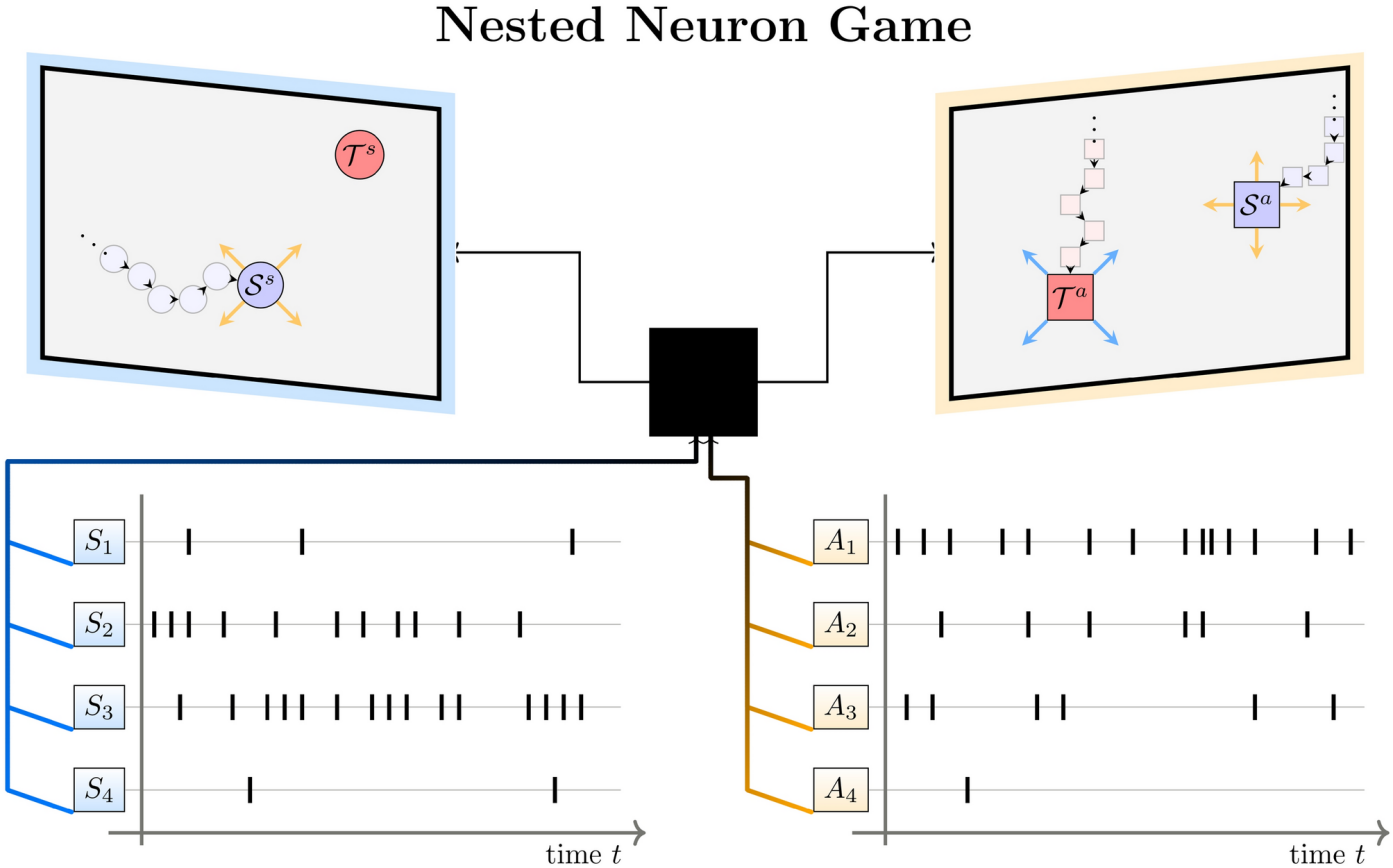
Experimental setup with sensory group (left) and motor group (right). Participants are split into designated sensory and motor groups of four people each and placed in front of either of two screens. The goal of each group is to steer a cursor into a target area, but only the target of the sensory group represents the externally given target, whereas the target of the actor group is determined by the activity of the sensor group. To achieve this target reach, each player has a push button and is assigned an unknown movement direction. Pressing a button results in a small displacement of the cursor on both screens due to the actor group’s actions and a small displacement of the target on the actor screen due to sensor players’ actions. Success in the Nested Neuron Game consists in (1) the actor group learning to reach the target set by the sensor group’s signal, (2) the sensor group learning to control the actor group and (3) the two groups coordinating and collaborating to steer the cursor towards the external target. Each button press is recorded resulting in eight spike trains, one for each player.

In our experiment illustrated in Fig. 1, an ensemble of eight players has to cooperate to solve a sensorimotor task that consists of steering a cursor sprite into a given target. The ensemble is split into two equally sized groups which we refer to as the sensor and the actor group. The sensor group can see both target and cursor, but cannot control the cursor. In contrast, the actor group can control the cursor, but cannot see it or the target. To close the loop, the sensor group can communicate with the actor group by sending a signal in form of another target sprite that is only shown to the actor group. Each player is equipped with a push-button. Whenever a sensor player presses their button, the target sprite on the actor screen moves in a predefined direction unknown and unobservable to the sensor player. Analogously, whenever an actor presses their button it causes a small displacement for both cursor sprites on both screens in two different predefined directions, also unknown to the actor (see Fig. 1 and section Experimental Methods for details).

Apart from the interdependence between groups, a major challenge of the game is the fact that all movement directions are initially unknown to the players and have to be learned on the fly, while all players can be active similar to neuron-like decision-making units. This setup constitutes a Nested Neuron Game, a hierarchical version of our previous experiment called the Neuron Game,^16^ as the actor group is playing a Neuron Game within the Neuron Game of the sensor group. During the game, we record button presses of all participants, which lets us draw conclusions about their sensorimotor learning progress and cooperation over time. To further explore learning and coordination, player-specific movement directions were randomized every six trials across 35 batches. Players’ behavior reflects increasing collaboration over time, as random actions alone cannot reliably achieve the goal of steering the cursor from start to target.

In what follows, we compare participants’ behavior to two different computational models. The first one is a Thompson sampling^51^ model that had previously been able to capture human behavior in a simple Neuron Game experiment by providing approximations to Bayes optimal decisions.^16^ In this model each player is assumed to hold and update a Bayesian belief about the consequences of pressing their button and to contribute to the group accordingly, leveraging the well-documented power of Bayesian integration and inference for efficient use of sensory information in uncertain environments^49,52,53^ (see section Model details). The second model consists of a bounded rational decision-network.^54^ The model treats the eight players as a two-layer network, in which the repeated communication between the sensor and the actor layer functions as a model for the game’s dynamics in which actors influence the sensor behavior, which in turn influences the actors’ behavior and so on (see section Model details). In this model, learning is modeled as decreasing noise in the task utility, so players can respond more and more precisely up to a certain limit. In both models, a major difficulty of the sensorimotor task is for decision-makers to keep track of the consequences of their own actions to infer knowledge from it, especially for the players of the sensor group. Interestingly, other models, including perceptron learning models and hierarchical feudal reinforcement models,^55^ were not able to solve the Nested Neuron Game.

### Credit assignment problem

In the Nested Neuron Game only the actors have immediate access to the consequences of their own actions on their own screen. Accordingly and in line with previous models of the Neuron Game,^16^ it is sufficient for actors to consider just the following time step when deciding whether their actions triggered an observable change. Although those observations might be noisy because of multiple decision-makers pressing their buttons simultaneously, over time these observations allow for growing certainty regarding the effect of their own actions, which results in actors improving their response to the signals sent by the sensor group.

Players in the sensor group on the other hand do not have immediate access to observations informing them about the consequences of their actions. Instead they have to consider longer sequences of observations after each of their button presses, as the consequences of their actions are only reflected in the responses of the actor group. These observations come with two main problems. The first problem faced by the sensor group is that the actors are not able to respond perfectly to their sensory input, especially in the beginning of each new trial. The second problem is that it is uncertain whether the (imperfect) response of the actors is a direct response to the action of a particular sensor player or of multiple sensor players that were active at the same time. To take into account the resulting delay in the feedback loop, we modified all computational models to allow for an extended sensitive period, such that players can learn over a prolonged time interval after they were active (see section Model details).

### Quantifying learning and collaboration within the groups

In order to assess learning and collaboration among the members of the sensor and the actor group, we analyze the decisions made by the groups as a whole and for each individual player within the respective group. To this end, we determine the group’s *improvement rate* as the ratio of group decisions that improved distance-to-target over the number of all group decision that were made. As this improvement rate is ignorant about individual contributions, we additionally investigate the *individual correct response rate* and the variance of stimuli distribution to which each player reacted. The individual correct response rate captures how often they contribute successfully to the group’s movement. The individual variance measures how specialized players are in their contribution. Moreover, we examine the internal consistency among the groups of players captured by the action time correlations between each distinct pair of spike trains generated by the players to describe to what degree the players act cooperatively. One would expect that players contributing similar skills, i.e. players with similar preferred movement direction, should tend to act together, whereas players with opposing displacement directions should refrain from acting simultaneously.

#### Group performance and learning

**Fig. 2:**
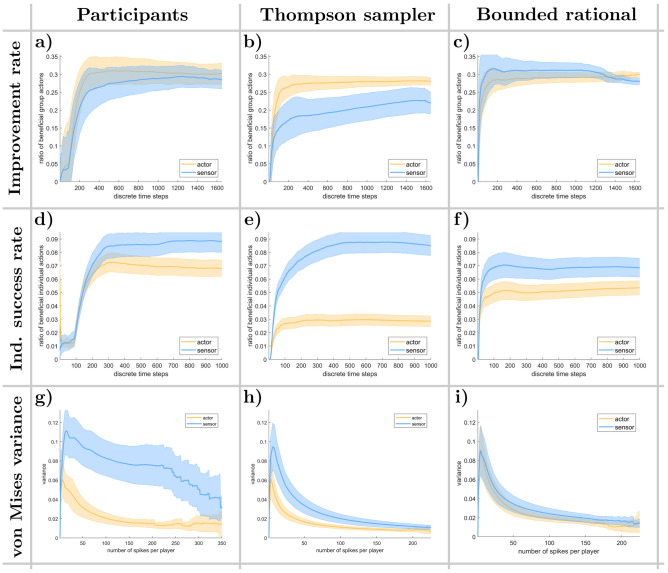
Group improvement, individual correct response rates and spike variance. (**a, b, c**) Proportion of time points where the cursor was moved closer to the target. (**d, e, f**) Individual correct response rates. A single player’s action is deemed to be a correct response, if it had steered the cursor towards the target independent of the remaining group members in both the sensor and actor group. (**g, h, i**) Time dependency of the variance of the spike triggered average. Shaded areas in all figures indicate 95% confidence intervals.

For this analysis, a *decision* of a single player corresponds to a player being active (i.e. pressing their button) or not within a time interval of approximately 125 ms (see Methods for details). A *group decision* refers to a vector containing the decisions of all players from the same group at a specific time and we analyze all decisions made over the course of multiple trials. A decision is called an *improvement*, if the new cursor position is closer to the (potentially new) target position than the previous cursor position. As displayed in Fig. 2a–c, the average improvement rate increases to approximately 30% within the first 200 time steps for the human participants for both the actor as well as the sensor group. This means that in 30% of the time steps an improvement occurs. The participants’ data exhibit a strong and statistically significant trend within the first 750 time steps for both the actor (Mann-Kendall test: $Z = 33.880$, $p < 10^{-50}$; Kendall’s tau: $\tau = 0.827$, $p < 10^{-50}$) and sensor group (Mann-Kendall test: $Z = 40.711$, $p < 10^{-50}$; Kendall’s tau: $\tau = 0.993$, $p < 10^{-50}$), suggesting a monotonically increasing relationship over time.

The bounded rationality model mimics this behavior with a slightly steeper performance increase. The Thompson sampling model reaches 25% and 20% improvement rate for the actor and sensor group, respectively. The most notable difference between the Thompson sampling model’s and human behavior is the difference in behavior between the actor and sensor group that remains even towards the end of the trial.

Similarly Fig. 2d–f show the average individual correct response rate of each decision-maker or player within the actor and sensor groups. A player’s button press (or absence thereof) counts as an individual correct response if the player’s action (or non-action) corresponds to the best possible choice in terms of target distance, independent of all other players. Note that it is possible to have an improvement without any individual response success, for example, an improvement for the sensor group when their cursor is moved towards their target position by the actor group without any sensor reacting correctly to the current target difference vector. Again, a test was conducted to examine whether a monotonic trend was present, revealing such a trend for the participants’ actor (Mann-Kendall test: $Z = 7.853$, $p = 3.997 \times 10^{-15}$; Kendall’s tau: $\tau = 0.192$, $p = 4.078 \times 10^{-15}$) and sensor group (Mann-Kendall test: $Z = 38.647$, $p < 10^{-50}$; Kendall’s tau: $\tau = 0.943$, $p < 10^{-50}$) as displayed in Fig. 2d.

In terms of individual success rate, the Thompson model matches the results of the human participants in the sensor group, but displays a considerable disparity between sensors and actors. The bounded rational model on the other hand, shows the same qualitative behavior as the human groups where the average actor has a slightly lower success rate than the average sensor.

Finally, Fig. 2g–i show the variance of a von Mises distribution that was fitted to the target difference vector angles to which the different players reacted. Over time, all players try to specialize their contribution to the movement of the cursor and adapt accordingly. These specializations eventually differentiate for each player to approximate the assigned displacement direction at the end of learning,^16^ causing the variance in target difference vector angles that trigger players’ responses to diminish. Note that a player’s decrease in variance should correlate with a higher individual correct response rate as long as it is centered around their assigned displacement direction. To assess whether this adaptation followed a consistent trend, we applied a Mann-Kendall test and determined Kendall’s tau, which confirmed a significant monotonic pattern in both the participants’ actor (Mann-Kendall test: $Z = -21.583$, $p < 10^{-50}$; Kendall’s tau: $\tau = -0.916$, $p < 10^{-50}$) and sensor group (Mann-Kendall test: $Z = -20.179$, $p < 10^{-50}$; Kendall’s tau: $\tau = -0.857$, $p < 10^{-50}$). While the actor groups of human participants and the two different models behave almost identically, in that the variance of stimuli to which they react converges rapidly to approximately $0.02\text{ rad}^2$, the human sensor group exhibits a much larger variance than the two models for almost all of the trials.

#### Group synergy

**Fig. 3:**
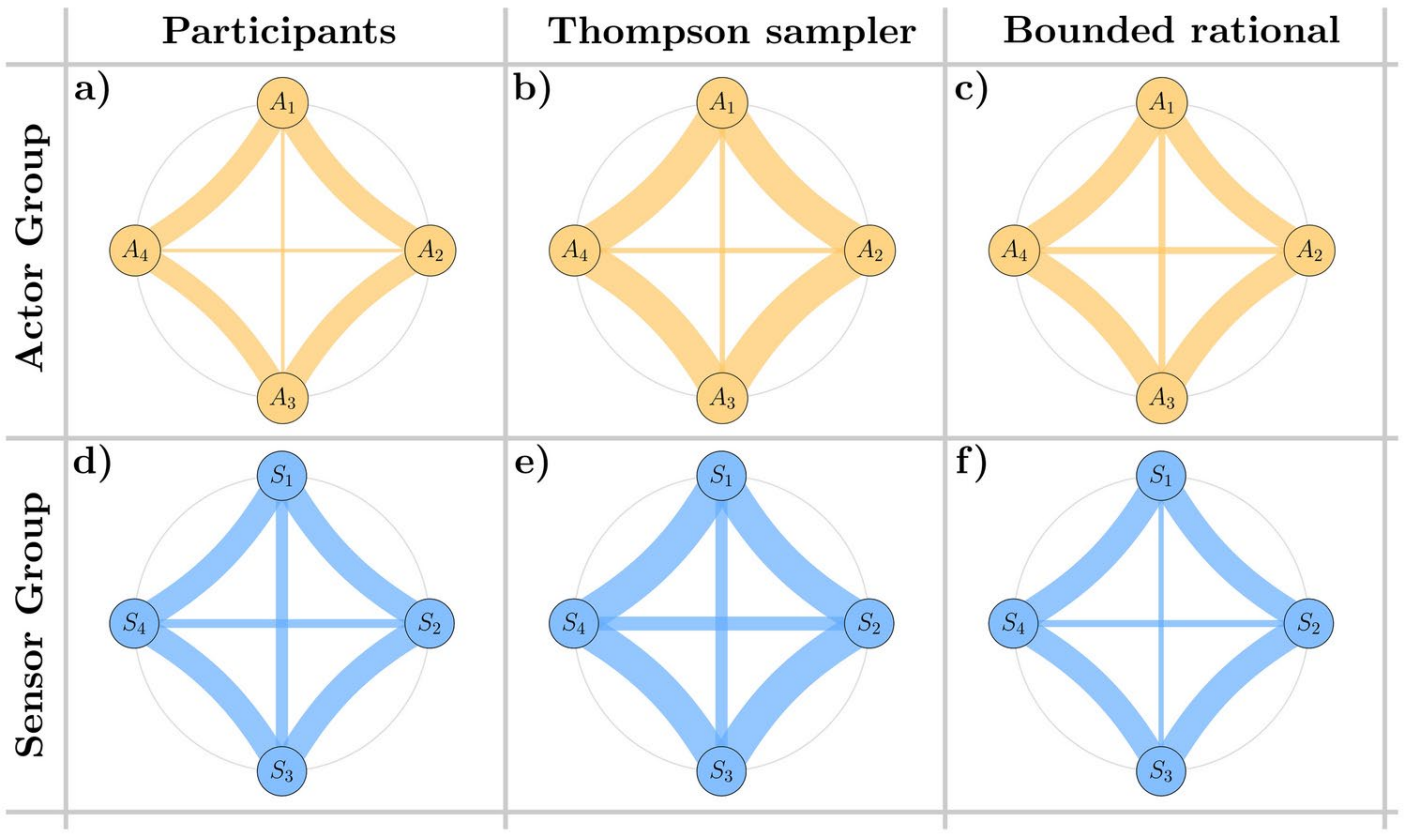
Action time correlation. In the chord diagrams, the thickness of the chords represents the correlation between players, following a linear scale. The thinnest chords (1 unit thick) correspond to a correlation of $-0.06$, while the thickest chords (20 units thick) correspond to a correlation of 0.13. (**a, b, c**) Action time correlation between all pairs of players in the actor layer for the human participants, the Thompson and the bounded rational model. (**d, e, f**) Action time correlation between all pairs of players in the sensor layer.

We can gauge the level of synergy in players’ interactions through the metric of action time correlation^16^ (see section Methods for details). This is calculated using the Spearman correlation between two binary vectors, indicating whether any pair of actors were active or inactive at a specific moment in time (see Table 2). Given that each group consists of four actor and four sensor players, we represent the correlation structure using chord diagrams. In these diagrams, the six unique player pairs within each group are depicted as chords, with the thickness of each chord indicating the strength of the correlation between the respective players’ actions. To enhance readability in Fig. 3, the players are arranged to mirror their neighborhood relationships. Based on the circular geometry of the displacement directions assigned to the players, we expect the strongest correlations to manifest as thick chords on the outer edges of the diagram, while the two chords in the middle remain thinner. This pattern reflects the tendency for neighboring players to act more in unison, whereas players controlling opposing displacement directions exhibit weaker correlations.

Figure 3a,d display the action time correlations for the human actor and sensor groups, respectively, averaged over all participating groups. Figure 3b,e, as well as Fig. 3c,f, show the corresponding correlations for the Thompson sampling model and the bounded rational model. The correlations among the subjects clearly display the anticipated neighborhood relationships, ranging from $-0.06$ for players with opposing movement directions to 0.08 for players with similar movement direction. Specifically, on average, the chords on the outer edges of the diagram are significantly thicker than those in the middle for both the actor group ($p = 2.488 \times 10^{-4}$) and the sensor group ($p = 1.067 \times 10^{-6}$). Furthermore, all models qualitatively replicate this pattern.

### Transfer of information between sensor and actor groups

Unlike a single layer network like the Neuron Game that focuses on learning and collaboration within a group sharing a common goal, the Nested Neuron Game investigates the cooperation of two distinct specialized groups, i.e. the sensor and actor groups, where sensors have direct access to the world but cannot act directly in the world, whereas actors are able to act in the world but can not determine the state of the world directly. Therefore, for successfully playing the game as an ensemble of players, it is necessary that (i) actors learn about their contribution to the overall movement and therefore are able to follow the sensor group’s commands, and (ii) sensors learn to transfer information about the actual goal to the actors to make them steer the cursor towards the target.

For both tasks the error signal received by any of the two groups at any point in time depends on the decisions made by players in their own group as well as decisions made by the other group. Crucially, the error signal may also depend on decisions made multiple time steps prior to the current time. For example, if a sensor player acts to respond to the position of the target, its own error signal changes a few time steps later in the future, when the actor players had time to react to the sensor player’s actions.

#### Ensemble coordination

**Fig. 4:**
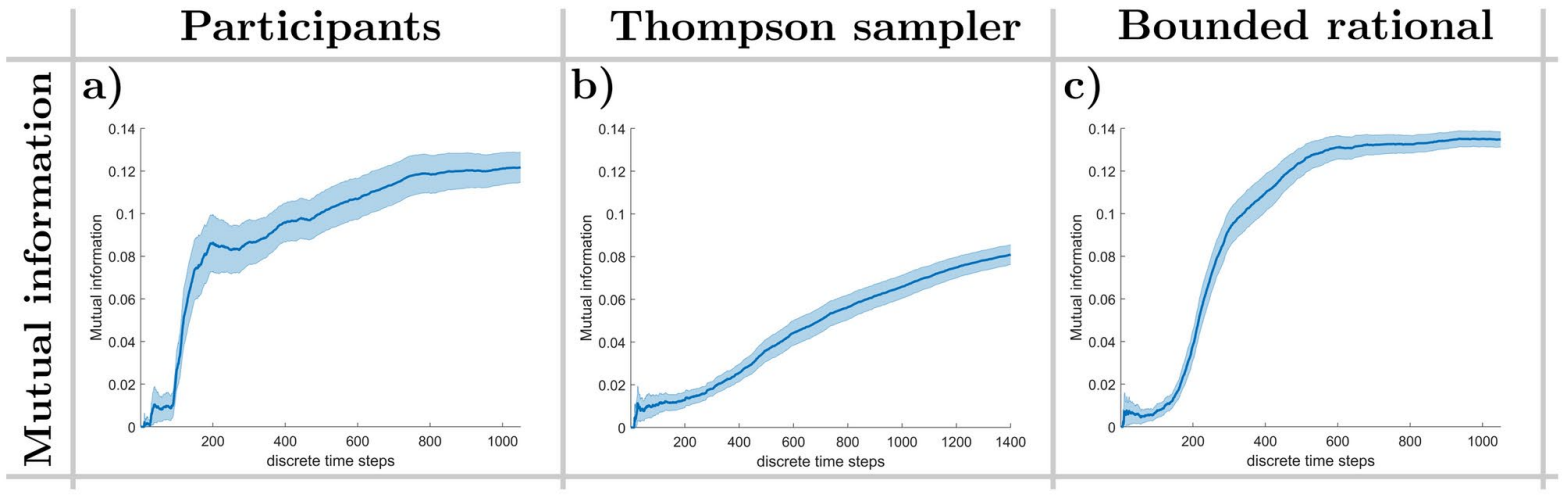
Mutual information, lag of maximum cross correlation. (**a, b, c**) Temporal evolution of the mutual information between the (discretized) target difference vector angle and the decisions of the actor group. Shaded areas indicate 95% confidence intervals.

In order to quantify how well the two subgroups of actors and sensors coordinate in the Nested Neuron Game, we analyze the amount of information obtained about the state of the world in the sensor layer by observing the actions taken by the actor layer and vice versa. To this end, we calculate the mutual information between the (discretized) target difference vector angle as seen by the sensor players and the decisions made by the actor group. Figure 4a–c illustrate that this mutual information starts out at almost 0 in the beginning of each batch of trials, stays at a low value for about 100 to 150 time steps and then rapidly increases up to a point where it flattens towards the later phases of the game. This firstly measures the time it takes the sensor group to learn how to control the actor layer to a reasonable degree and secondly describes how well the sensor group is able to communicate the target difference vector to the actors in the later stages. A Mann-Kendall test confirms that this increase follows a significant monotonic trend in the participant group (Mann-Kendall: $Z = 39.376, p < 10^{-50}$). The strength of the monotonic relationship is further supported by Kendall’s tau ($\tau = 0.961, p < 10^{-50}$), indicating a strong positive correlation. Figure 4b shows that the Thompson model performs worse than the human participants in that the increase in mutual information is much slower.

Furthermore, as stated above, the error signal received by the sensor players has a certain delay, as the actors must first react to the signals sent by the sensors. Conversely, once the actors’ displacements are observed by the sensors, they must respond by sending signals that either correct the movement or reinforce the same direction. To assess the delay emerging from this continuous exchange, we calculated the cross-correlation between decisions made by actor and sensor players, identifying the lag corresponding to the maximum correlation as an indicator of when one group’s actions prompt a response in the other. For human participants, this maximum lag in the sensor-to-actor correlation ranged between 4 and 5 time steps, corresponding to 500 to 625 ms. In simulations, the Thompson sampling model exhibited maximum lags between 2 and 4 time steps, whereas the bounded rational model showed a consistent lag of approximately 4 time steps.

### New challenges in the nested neuron game

Unlike the previous Neuron Game experiment,^16^ where all participants received the same input and reacted together to solve the sensorimotor task, the Nested Neuron Game splits the participants into two groups that receive different input depending on their role of being either part of the sensor or actor group. Both groups can be thought of as playing a game similar to the original Neuron Game, where (i) the actor group plays a Neuron Game with a more complicated movement task as their target is moving, and (ii) the sensor group plays a Neuron Game with a more challenging learning task as they cannot observe the consequences of their actions directly, but must infer consequences of their actions over a longer period of time from a very noisy signal.

**Fig. 5:**
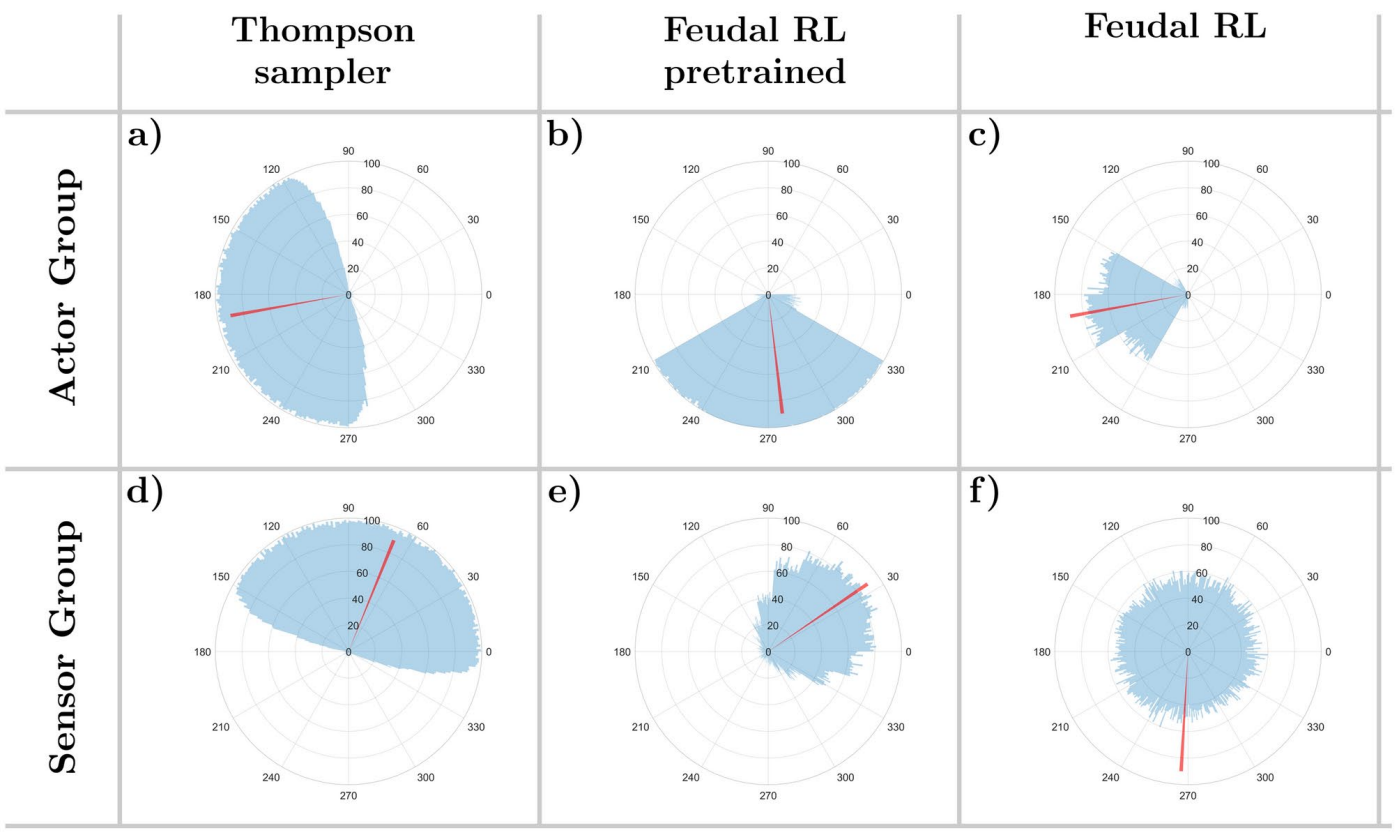
Circular histograms of agent activity in response to target difference vector angles. Each agent was presented with 72,000 stimuli, uniformly distributed across all directions. The plots display polar histograms of the stimuli that elicited a response from the agents. Each bin represents a 1-degree interval, and the histogram height is scaled such that a value of 100 corresponds to the agent reacting with a button press to 100% of stimuli in that bin. Red indicators mark the assigned displacement direction for each unit. (**a, d**) Agents from a Thompson sampling learner after playing the Nested Neuron Game. (**b, e**) (Feudal) reinforcement learning agents pretrained with 150 noiseless signals, which successfully played the Nested Neuron Game and continued learning during gameplay (**c, f**) Reinforcement learning agents without pretraining that failed the game.

Intriguingly, these additional challenges could not be tackled by a number of learning algorithms that are able to solve the simple one-layer Neuron Game, even if we modified these methods to include an adaptive learning rate to deal with delayed action consequences (see Methods for details). In particular, the RL and the ANN model, consisting of a reinforcement learning model and a model comprised of perceptron learners^16^ were unable to solve the Nested Neuron Game. The main problem the learners could not overcome was the more challenging learning task in the sensor layer (c.f Fig. 5).

#### Noisy signals and uncertain rewards

The multi-agent nature of the Nested Neuron Game and the inherent inability to distinguish between consequences produced by the different agents, especially in the sensor layer, lead to several learning models failing the Nested Neuron Game. Figure 5 illustrates the difference between a trained Thompson sampling agent and a reinforcement learning agent that is able to solve a one-layer Neuron Game, but not the two-layer Nested Neuron Game. The figures show polar histograms of stimuli vectors that incite the decision-making units to be active (i.e. players to press their buttons in the case of human participants). The actor and sensor agents in the Thompson sampling model, as displayed in Fig. 5a,d, manage to learn about their assigned displacement direction and are able to react almost perfectly to all presented stimuli. The Reinforcement Learning agents on the other hand were not able to solve the game. Figure 5c shows that, despite the overall failure to complete the game, the actor group was still able to correctly learn about their contribution to the actual movement of the cursor (which is in line with the findings of the previous study were the same reinforcement agents solved the easier game). The main reason for the failure is that the sensor learners are not able to identify their assigned displacement direction, leading them to react to almost all stimuli at chance level (c.f. Fig. 5f). To investigate whether or not the highly noisy reward signals are the main cause of this problem, we pretrained the same reinforcement learning models with 150 observations ($\approx 10-15\%$ of observations made during the Nested Neuron Game) together with a noiseless reward signal, where the actor group always reacts perfectly to the sensory signal (c.f. Fig. 5b,e). In this simplified scenario, the reinforcement learners are able to correctly learn their assigned movement directions in the sensor group and successfully master playing the Nested Neuron Game almost on par with the Thompson learners.

#### Number of units and layers within the ensemble

Another challenge faced by both the participants and all the models lies in the fact that each layer in the Nested Neuron Game only contained four players. When layers consist of more players, as in the one-layer game with eight players, the group is able to solve the game even if one or two players fail to learn their assigned movement direction. In contrast, the smaller groups in the Nested Neuron Game are more susceptible to errors, as every single unit is critical for successfully steering the cursor towards the target. This raises the question whether larger layers with more units or networks with more than two layers would perform better than the models discussed so far?

To explore this question theoretically, we conducted simulations modifying the Nested Neuron Game to allow for arbitrary network structures by varying both the number of layers and the number of units per layer. This investigation was motivated by the hypothesis that expanding the size and complexity of the network might enhance its performance. Such an exploration aligns with the conceptual framework of the experiment as an analogy for a small brain-like structure, where specialized sub-structures interact to achieve coordinated outcomes.

**Fig. 6:**
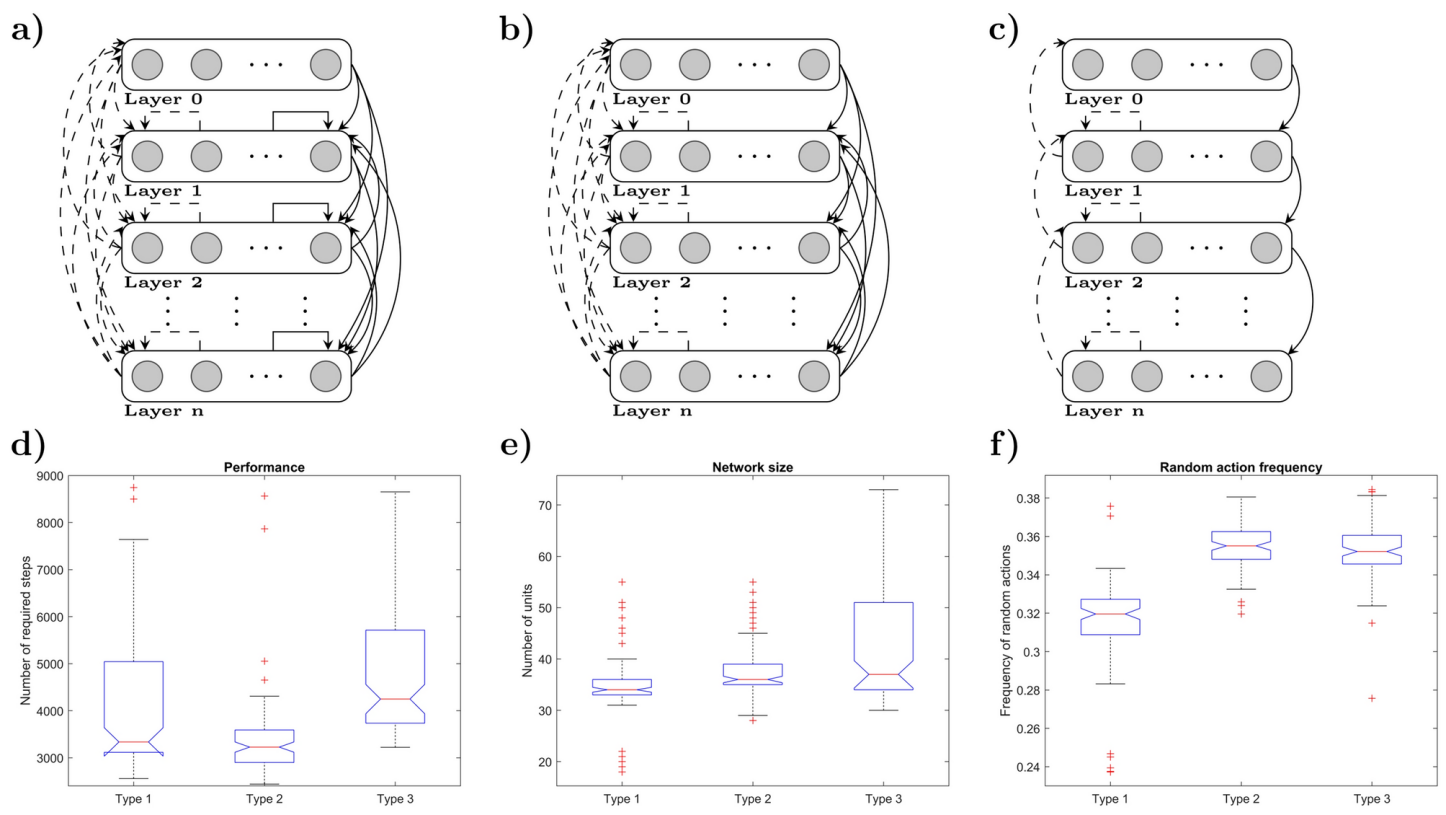
Multilayer networks. Networks evolved with the following restrictions: **a** Scenario 1: All connections between the layers are allowed. The target (reference value) for Layer 0 is fixed and Layer 0 can not move its own cursor. (**b**) Scenario 2: Same as Scenario 1 with the additional restriction that no layer can change its own target. (**c**) Scenario 3: Feedforward layers. Each layer $L_i$ can only influence the target of $L_{i+1}$. Conversely, each layer $L_{i+1}$ can only move its own state and the state of $L_{i}$. (**d**) Performance of the 65th generation measured by the number of steps required to successfully complete the Nested Neuron Game ten times. The box plot shows the performance of 100 learning systems in the 65th generation. (**e**) The total number of decision-making units in systems of the 65th generation. (**f**) The average value of the parameter describing the frequency of random actions as observed in the 100 learning systems of the 65th generation.

Using an evolutionary approach,^56,57^ we optimized the network architecture starting from an initial design. The number of layers, the number of agents per layer, the weight matrices, and the degree of stochasticity employed by the units were allowed to mutate. Throughout the optimization, it was ensured that the sensor layer’s externally given target could not be moved by any agent and that the sensor layer agents could not change their own position. We employed the Thompson sampling model as the base decision-making model for each agent, with all other hyperparameters kept fixed. To understand the influence of structural constraints, we investigated three different sets of restrictions on the permissible network structures:

- Scenario 1: Units could influence arbitrary layers and move the targets of other layers, including targets within their own layer (except for the sensory layer). Compare Fig. 6a.
- Scenario 2: Same as Scenario 1, but agents were not allowed to move targets within their own layer. Compare Fig. 6b.
- Scenario 3: Same as Scenario 2, but influence on other layers was restricted to operate strictly sequentially. Compare Fig. 6c.

The evolutionary optimization aimed to maximize the performance of the network by minimizing the number of steps required to solve ten instances of the Nested Neuron Game. The optimization process had to balance increasing the number of agents and the degree of stochasticity against the resulting increase in system complexity. While more agents can enhance movement per time step, they also complicate the learning problem. Similarly, greater stochasticity increases exploration but can lead to uncontrollable random behavior.

Figure 6 illustrates the main results of this evolutionary process. After 65 generations, the median time required by the learning systems decreased from approximately 25,000 time steps to 3,300 for Scenarios 1 and 2, and to about 4500 for the sequential Scenario 3 (see Fig. 6d and Supplementary Figures S1–S3 for further details). For all scenarios, the median learning system in the 65th generation consisted of approximately 35 units (Fig. 6e), distributed across an average of 3.0, 3.1, and 2.6 layers for Scenarios 1, 2, and 3, respectively. These findings suggest that even for a relatively simple task, a more complex network architecture provides performance benefits compared to a two-layer network.

Additionally, as depicted in Fig. 6f (and Supplementary Figure S2), an increase in random behavior among individual agents improved the overall system’s performance. This implies that high-noise signals from a larger number of units are more beneficial than low-noise signals from a smaller pool of actors. These results highlight how an evolutionary framework can provide valuable insights into the relationship between network architecture and task performance, further supporting the analogy of our experiment to small brain-like structures with specialized sub-units.

## Discussion

In our study, we explore a novel experimental paradigm where two groups of human participants have to work together to solve a complex sensorimotor task. Each participant assumes the position of a decision-maker who makes binary choices in response to a visual stimulus that is shared among players that belong to the same specialized group. We evaluate the extent to which these individual neuron-like participants with binary action space cooperate, scrutinize their progression in learning and joint performance, and compare the human group behavior observed in the experiment to different computational models, in particular a Thompson sampling model that simulates the behavior of the human participants by approximating Bayes optimal decisions, and a bounded rational decision-making model that mimics the learning process by a annealing the amount of noise in the decision-making system.

The Thompson model shows that a collection of agents, which are only able to adapt to locally available information, can achieve a complex sensorimotor task without any of the agents actually knowing their true contribution to the whole system. Unlike traditional hierarchical systems, where one layer makes a plan for a specific movement and gives instructions to a lower layer that then reacts accordingly and is subsequently rewarded by the higher layer (such as in Feudal Reinforcement Learning^55^), the units in the Thompson model interact at the same time scale, forming a symbiotic nested control loop where each group can operate as a unit. This allows the Thompson model to be easily scaled to systems with many more agents that form networks with many layers, as shown in the previous section.

The bounded rational model, on the other hand, although often closer to human performance in the experiment, is merely providing a descriptive model and is expensive to simulate. It provides a description of the ensemble of agents as a highly connected, loopy network of bounded rational binary decision-makers, that operate optimally given their limitations.^54^ The model can be adjusted by tuning the parameters of the involved units, making the bounded rational model a powerful tool when it comes to classifying *how well* a different model is solving the sensorimotor task.

As a third class of models to explain intergroup coordination in the context of the Nested Neuron Game, we have considered hierarchical reinforcement learning models^58–60^ and found clear limitations. Specifically, reinforcement learning agents in the sensor layer struggle to handle the dual challenges of noisy signals—arising from the imperfect actions of the actor group—and delayed rewards, as beneficial states are often reached only after several intermediary steps. These findings align with prior research indicating that standard model-free reinforcement learning approaches are less effective in tasks with sparse or delayed rewards and high environmental uncertainty.^16,61–64^ Moreover, hierarchical reinforcement learning designs usually require a separation of temporal time scales on the different levels of the hierarchy. For example, in feudal reinforcement learning the managers of the higher level stay idle until the workers of the lower level have finished. In our case, this would correspond to each level playing a Neuron Game, where the lower-level actor group would play a full Neuron Game to the end for each command of the higher-level sensor group. In contrast, the nested Neuron Game that we investigated requires concurrent optimization, where the actor group is chasing a moving target and the sensor group struggles with (initially) random controls. While reinforcement learning with moving targets is notoriously difficult,^65,66^ in our case this scenario is exacerbated by the dynamic coupling of the moving target to the reaction of the sensor group.

In contrast, models that maintain an internal representation of the consequences of their binary actions, such as the Thompson sampling model, demonstrate a distinct advantage. This is consistent with evidence suggesting that agents capable of anticipating the outcomes of their own actions-and, in collaborative tasks, the actions of their partners-are better equipped to coordinate effectively in uncertain environments.^67–71^ Notably, in our experiment, the Thompson model’s sensor group successfully navigated the task despite the inherent uncertainty, learning to lead the actor group effectively. This outcome reinforces the importance of incorporating predictive mechanisms and internal models in hierarchical systems, particularly for agents responsible for decision-making under noisy conditions.

The differences between the sensor and actor groups further illustrate the challenges of hierarchical reinforcement learning. While the actor group in all models was generally successful in learning its preferred direction, the sensor group in the reinforcement learning-based models failed to learn effectively. This failure is particularly pronounced due to the cascading impact of sensor group errors: the inability to generate meaningful guidance for the actor group caused the entire hierarchical system to collapse into random behavior. This aligns with findings from the hierarchical reinforcement learning literature, which emphasize that top-level agents in a hierarchical framework often require more sophisticated mechanisms to manage the complexity of planning and learning in uncertain or noisy environments.

Our findings underscore the importance of designing hierarchical learning frameworks that can handle uncertainty and delay in reward signals, particularly for high-level decision-makers. By showing that the success of the Thompson sampling model hinges on its ability to predict outcomes and adjust to noise, we provide evidence supporting the utility of predictive, model-based approaches in hierarchical and collaborative settings. These insights contribute to ongoing discussions in reinforcement learning research, highlighting the limitations of model-free methods in complex, hierarchical coordination tasks and suggesting avenues for improving agent design in such systems.

### Leading and following—when groups set the target of other groups

The concept of a hierarchy of agents that depend on one another has been examined across diverse environments and frameworks.^60,72^ One such framework is feudal reinforcement learning,^55,73^ where the learning process of the agents involved is structured hierarchically, with higher-level controllers assigning tasks to lower-level agents. In contrast to our experimental setup, were sensor and actor players act on the same timescale, higher-level controllers in feudal reinforcement learning set overarching goals, wait for the lower-level agents to deal with their respective tasks and provide reward to the lower-level agents according to some measure of how well they achieved the goals set by the higher-level controller. A stricter temporal separation between the signals sent by the sensor group and the actions of the actor group following these signals might allow hierarchical reinforcement learning algorithms to succeed in our task. Crucially, however, they are not able to reproduce the concurrent optimization process observed between our sensor and actor groups.

The setup of one group leading and one group following has also been previously investigated in the context of economic game theory, especially in the Stackelberg leadership model,^74,75^ where in the context of a duopoly a leader (firm) moves first, for example by setting an output or a price for some good, while another follower (firm) subsequently adjusts its responses to react to the actions of the leader. The analogy to the Nested Neuron Game lies in how the Stackelberg model is solved by backward induction, where the leader considers the follower’s optimal reaction, anticipating how the follower will adjust once it observers the leader’s actions. This relates to how the sensor group learns about how the actor group will react to signals sent by the sensor group, leading to a similar kind of interaction, where the leaders or sensors lead by choosing an action based on how they anticipate the other group will react to it and the followers or actors reacting to the quantity or target set by the sensors. This principle has also been proposed for cooperative control of robots, where multiple units need to make decisions that are consistent with a shared goal.^76–78^

Another domain where the principle of interdependent layers setting goals for each other proves to be beneficial is artificial neural networks. There, target propagation has been proposed as a promising alternative to back-propagation to perform credit assignment in networks.^79–81^ In contrast to back-propagation,^82^ which adjusts weights based on gradients computed from the output error, target propagation sets targets for each layer based on the layer above. The idea is to propagate “targets” instead of “errors” down the network such that each layer modifies its parameters to make its actual output closer to the target provided by the layer above. This approach can be seen as a form of local learning, where each layer learns independently of the others.^83^ This is similar in nature to how the different layers in the Nested Neuron Game and its extensions to multilayer setups work, where all layers can be thought of as independent systems that each play their own modified version of the Neuron Game. More generally, each layer independently solves a control problem that is defined by a reference value or target set by other layers and disturbances created by other players and agents. Notably, there is no communication between the different agents of a layer or across layers required beyond the input of targets and (potential) disturbances. Neither is it necessary for any unit to understand what its neighbors do nor is it necessary for any unit to understand what happens in another layer. The system as a whole functions as long as all local tasks can be fulfilled.

### Global collaboration, but everybody controls only their own perception

It is in this image of small units that fulfill a local task unaware of their role in a potentially complex system that connects the Nested Neuron Game to broader biological concepts. One such concept, for example, is homeostasis, which describes how the state of internal, physical, and chemical conditions are maintained by living systems.^84,85^ There, the equilibrium is the condition of optimal functioning for the organism that includes many variables, such as body temperature and fluid balance,^86^ being kept within certain pre-set limits. Similar to the Nested Neuron Game, where each neuron performs its function without understanding the larger picture, cells in an organism carry out their individual tasks. The idea that homeostatic loops could be nested in a hierarchical fashion to achieve complex behavior has been previously suggested by cyberneticists like Ashby.^87^

One prominent framework that has arisen from this point of view is perceptual control theory, which offers a powerful lens for understanding behavior, emphasizing the continuous process by which individuals compare actual perceptions to desired perceptions and adjust their actions accordingly to minimize discrepancies.^41,43,88^ This framework, rooted in cybernetics^89^ and extended by Powers and Bernstein,^46,90^ has been applied across domains to explain how hierarchical control systems can maintain desired states while adapting to environmental changes. A hierarchical perceptual control system organizes control loops into nested levels, with higher levels setting reference values for subordinate levels, allowing for a structured division of labor. At the top of this hierarchy, abstract goals guide overall behavior, while lower levels focus on more specific perceptual and motor adjustments, with bidirectional information flow enabling refinement and coordination.

In this context, the Nested Neuron Game provides an experimental analog to a small hierarchical perceptual control system. The sensor layer in our setup mirrors the top level of a perceptual control hierarchy, directly observing the target (goal) and setting reference values for lower levels. The actor units beneath them adjust their actions in response to these reference values, focusing only on their local perception of the target difference vector. This decentralized structure captures key aspects of perceptual control theory by demonstrating how coordination emerges, as each actor independently minimizes their perceptual error.

Our findings align with core principles of perceptual control theory while extending them into a novel experimental domain. Specifically, our evolutionary simulations described in section Results highlight the benefits of expanding hierarchical network structures to include additional layers and actors. Larger pools of decentralized agents, each operating independently but as part of a broader system, were shown to enhance performance through increased redundancy and stochasticity, aligning with the perceptual control theory principle that variability at lower levels can support stability at higher levels. These results suggest that hierarchical perceptual systems may benefit from growth and complexity under certain conditions, even when individual units lack global awareness.

By applying perceptual control theory to our experimental paradigm, we not only demonstrate its applicability to human group behavior but also provide insights into the advantages and limitations of hierarchical control systems in decentralized environments. This work complements existing research on perceptual control by showing how specialized substructures within a larger system can achieve coordinated outcomes without requiring agents to have explicit knowledge of the overall goal or the state of other units, underlining how complex systems can achieve robust coordination through local control processes.

### Evolution of cooperation and specialization

The evolution of cooperation has been extensively explored in evolutionary and theoretical biology, encompassing mathematical principles at both the molecular and organismal levels.^91,92^ Various models, including kin selection and reciprocity, have been proposed to explain cooperative behavior across different systems, be it on a cellular level or in humans. Analogously, specialization evolved across various scales alongside cooperation being a crucial part of the adaption process of different systems both in a biological as well as a sociological sense. On an abstract level, we may ask about the principles that govern cooperation within systems that involve multiple specialized decision-making units. This examination involves exploring collective learning and the dynamics between individual and group decision-making. Therefore, our results and methods presented in this study may also be of interest to the broader research on cooperation in groups.

## Conclusion

In summary, we introduced a novel experimental paradigm to explore sensorimotor coordination between groups of human players that take on specialized roles to achieve an overarching goal. Each individual in each group functions as a neuron-like binary decision-maker, determining whether to be active or not at any given moment. Compared to the number of individual neurons in the brain, the number of players in our game is certainly limited, and it would be great in the future to test much larger groups, possibly in an online setting. Here we have tried to address this limitation at least in part, by running simulations of our model for more complex networks. From the different learning models we compared to human group behavior, a learning rate-adjusted Bayesian decision-maker was able to complete the Nested Neuron Game, creating a nested system of agents, each trying to contribute to a shared control task. An information-theoretic bounded rationality model could also mimic the observed behavior, but was limited in that it is only a descriptive model and not a process model. While hierarchical reinforcement learning models failed to complete the task, as such models typically assume a separation in time scales, we cannot conclude that there may not be more complex models that would allow for concurrent co-optimization as observed in our experiment. Another open question for the future is, whether our setup could generalize to more complex motor coordination tasks and whether groups of humans could solve such tasks beyond simple target reaching.

## Methods

### Experimental methods

#### Participants

Thirty-two students (eleven female, twenty-one male) from Ulm University participated in the experiment. Recruitment was conducted via flyers distributed on campus, and participation was open to all students, as the task only required viewing a screen and pressing a button. Participants were assigned to four groups of eight, with each group further divided into two subgroups of four. The experiment lasted approximately four hours, including two breaks, and participants were compensated with 40€ (10€ per hour). The purpose of the study was not disclosed to participants beforehand. After the experiment, they were fully debriefed and informed about the study’s objectives.

#### Ethics statement

All participants gave informed consent, and the study was approved by the ethics committee of Ulm University (protocol number 210/16). All experiments and methods were performed in accordance with the relevant guidelines and regulations.

#### Setup

For each experiment, eight subjects took part in the Nested Neuron Game, a computer game adapted from the Neuron Game that was originally developed at the Bernstein Center of the Albert Ludwigs University of Freiburg. Each participant employed a push-button controlled by an Arduino Leonardo Microcontroller.

At the start of the experiment, the group of eight subjects was split into two equally sized groups of four dubbed sensor group and actor group. The subjects were arranged in front of two separate computer screens such that the sensor group was not able to see the actor group’s screen and vice versa. All participants were instructed to conceal their buttons from other players’ views. To eliminate auditory cues from button clicks made by other players, participants were provided with headphones or earplugs. Additionally, a soundtrack containing rain sounds and white noise played from a speaker positioned behind the participants to ensure that only visual responses from their respective screens served as cues for evaluating the effects of each player’s actions.

The players in the different groups were not told how the groups and their button presses interacted. Instead, they were told that the purpose of the experiment was to find out if a group with easy learning but complicated target reaching task (actor group) had better or worse performance than a group with complicated learning but easy target reaching task (sensor group). For this the actor screen also displayed points and between two consecutive trials, i.e. whenever the sensor group reached their goal, the points of the actor and sensor group were displayed on both screens. The points of the actor group corresponded to the number of frames for which their cursor and their target sprite touched and the points for the sensor group were determined by adding or subtracting a random number from the actor group’s points. The point system served no other purpose than keeping up players’ motivation.

#### Experimental design

Each trial in the Nested Neuron Game consists of two groups of four players that have to cooperate to steer a cursor from an initial starting position into a circular target area. One of the groups, namely the sensor group, has direct access to the position of the cursor and the position of the target displayed on their screen, whereas the actor group does not have direct access to either of those positions. The actor group, on the other hand, is the group that is able to move the cursor. In contrast, the sensor group does not have any direct influence on the movement of the cursor, but can only send “instructions” to the actor group’s screen in the form of a moving target that the actor group is trying to catch with their own cursor. This way, the actor group simultaneously moves their own cursor displayed on their own screen and the cursor displayed on the screen of the sensory group that the actor group cannot see. The goal of the game for the sensor group is to learn how to control the actor group and then to instruct the actor group on how to move the cursor to the target. The goal of the actor group is to learn to react correctly to the signal send by the sensor group and then to act according to the sensor group’s commands.

Each player in each group is assigned an unknown movement direction angle specific for that player. If a sensory player presses their button, there is no change at all visible on their screen, but the target on the actor screen is moved in that direction. If one of the actor players presses their button, the cursors on both screens are displaced according to that player’s assigned movement direction. The movement directions were randomized every 6 trials and each player was randomly assigned one out of four approximately equiangular directions of two rotated windroses, such that the displacement directions of the sensor players never coincided with the movement directions of the actor group. The target and start positions on the sensor layer were randomized at the beginning of every trial and were given by a uniformly sampled starting position and an angle that was chosen from a set of six approximately equiangular angles for each batch of six trials. The center of the target area was then placed at a fixed distance 840 pixels away from the starting position. For the actor layer the cursor and target were placed at the center of the screen, where the moving target was invisible for the first few time steps.

At the end of every successful trial, the trajectories of both cursors, the trajectory of the center of the target area on the actor group’s screen and the action times (in milliseconds) of all players were saved and the game was paused. Before every trial there was a three second countdown and whenever the movement directions were randomized (once at the start of each batch of six trials) there was a flashing blue and orange screen and a note that informed the participants that the movement directions were randomized. Each group of participants completed 5 sets of 7 batches consisting of 6 trials each. This means each group carried out 210 successful trails over the course of approximately 4 hours with two breaks after the second and fourth set.

### Data collection

We recorded the button presses of all participants throughout the task. Each button press was time-stamped and associated with the corresponding player, allowing us to analyze individual and group actions (Table 1). These recordings enabled us to classify actions as beneficial or detrimental, quantify cooperation within subgroups through pairwise correlations, and evaluate information transmission between groups using mutual information (see below for details).

**Table 1:** Summary of experimental design.

	Description
Participants	32 participants, split into four groups of 8, each further divided into two subgroups: sensors and actors
Independent variables	**Role**: Sensor vs. Actor **Time**: Early, middle and late stages of the game
Dependent variables	$\cdot$ Correctness of actions (correct vs. incorrect button presses) $\cdot$ Pairwise correlation of button press vectors with subgroups (cooperation) $\cdot$ Mutual information between target direction (sensor group) and action (actor group) (information transmission)
Data collection	Button presses recorded for all participants, time-stamped and represented as binary vectors
Primary outcomes	$\cdot$ Learning effects (measured as an increase in beneficial actions and a decrease in the variance of action-triggering stimuli) $\cdot$ Cooperation (via pairwise correlations) and information transmission (via mutual information)
Task description	The Nested Neuron Game presents a hierarchical task, where sensors observe the environment and send signals to actors, who then act accordingly. Each participant must figure out their contribution to the task with each button press, all while working collaboratively toward a shared goal, without explicit verbal or physical communication

### Data analysis

#### Time discretization

For our data analysis, we discretize time in the experiment by finding the longest time interval of length *L* such that two successive button presses of individual players are at least a time *L* apart. Accordingly, we treat events that occur within the same time interval of approximately 130 ms as if they occurred at the same point in discrete time. Table 2 summarizes the lengths of the time intervals chosen for the four different groups of participants.

**Table 2:** Time intervals lengths for the different groups of participants.

	Group 1	Group 2	Group 3	Group 4
Length	130 ms	129 ms	125 ms	125 ms

#### Notation

In this study, we investigate the interaction of two coupled groups of decision-makers who are connected through their observations and rewards and must learn to cooperate in order to accomplish a sensorimotor task. The (sub-) task of each group is to move a cursor to a target, where the actions of individual agents have different consequences, which can be summarized as follows. With *s* and *a* being the names of the sensor and actor group, the following transition model applies to the position of the cursors $S^s$ and $S^a$, i.e., the state of the individual groups:

1 $$\begin{aligned} S^s_{t+1}&= f^s_{{\text {cursor}}}(S^s_t, A^a_t) \end{aligned}$$

2 $$\begin{aligned} S^a_{t+1}&= f^a_{{\text {cursor}}}(S^a_t, A^a_t) \end{aligned}$$

where the group action $A^g_t = (A^g_{t,1},\ldots ,A^g_{t,4}) \in \{0,1\}^4$ is a vector that contains the binary actions (active, inactive) taken by members of group $g \in \{s,a\}$ at time *t*. The functions $f^s_{\text {cursor}}$ and $f^a_{\text {cursor}}$ map previous cursor positions and actions from actor group players onto new positions with a population vector method, such that

3 $$\begin{aligned} f^s_{{\text {cursor}}}(S^s_t, A^a_t)&= S^s_t + \alpha \sum _{i=1}^4 A^a_{t,i} \mu ^a_i \end{aligned}$$

4 $$\begin{aligned} f^a_{{\text {cursor}}}(S^a_t, A^a_t)&= S^a_t + \beta \sum _{i=1}^4 A^a_{t,i} \mu ^a_i \end{aligned}$$

where $\mu ^a_i$ is the assigned movement direction for actor *i*. Players are initially unaware of their assigned movement direction and have to estimate it from the total displacement caused by the entire group. The parameters $\alpha$ and $\beta$ fix the step size.

Similarly, the transition model for their respective targets, $T^s$ and $T^a$, can be written as

5 $$\begin{aligned} T^s_{t+1}&= T^s = \texttt{constant} \end{aligned}$$

6 $$\begin{aligned} T^a_{t+1}&= f^a_\text {target}(T^a_t, A^s_t) \end{aligned}$$

where the constant target $T^s$ is externally given and specifies the sensorimotor task for the whole ensemble of players. The function $f^a_{\text {target}}$ maps previous target positions and actions taken by members of the sensor group onto new target positions:

7 $$\begin{aligned} f^a_{\text {target}}(T^a_t, A^s_t)&= T^a_t + \gamma \sum _{i=1}^4 A^s_{t,i} \mu ^s_i \end{aligned}$$

where $\mu ^s_i$ corresponds to the assigned movement direction for sensory player *i*. Again these assigned movement directions have to be learned over time. The parameter $\gamma$ determines the step size.

As each individual in every group decides about whether or not to press the button at every point in discrete time, the data collected from the recordings of the experiment is of the following form:

- ${\textbf{S}}^s = \left( S^s_t\right) _{t \in 1,\ldots ,N}$ and ${\textbf{S}}^a = \left( S^a_t\right) _{t \in 1,\ldots ,N}$ are $N \times 2$ matrices. Each row $S_t$ consists of the *x*- and *y*-coordinates of the respective cursor at time *t*.
- ${\textbf{T}}^a = \left( T^a_t\right) _{t \in 1,\ldots ,N}$ is a $N \times 2$ matrix. Each row ${T}^a_t$ consists of the *x*- and *y*-coordinates of the actor target at time *t*. ${\textbf{T}}^s = \left( T^s_t\right) _{t \in 1,\ldots , N}$ are the constant coordinates $T^s_t = T^s \,\, \forall t$ of the sensor target.
- ${\textbf{A}}^s = \left( A^s_t \in \{0,1\}^4\right) _{t \in 1,\ldots ,N}$ and ${\textbf{A}}^a = \left( A^a_t \in \{0,1\}^4\right) _{t \in 1,\ldots ,N}$ are $N \times 4$ matrices that represent the button presses or spikes of the groups.

The relevant stimulus to which decision-makers respond is the angle of the target difference vector $\psi _t=\measuredangle (S_t,T_t)$, which is calculated from the positions of the cursors $S_t$ and targets $T_t$ at every point in discrete time *t*. We use the function $\phi$ to translate from an angle to a unit vector of the same direction:

$$\begin{aligned} \phi \Big (\measuredangle (S,T)\Big ) = \frac{S-T}{\Vert S-T\Vert } \end{aligned}$$

#### Improvement rate and individual correct response rates

The improvement rate of group $g \in \{s, a\}$ is determined by how often the distance to the target is reduced from one step to the next. Formally, group *g*’s improvement rate $R_T^g$ for a trajectory of length *T* is defined as

$$\begin{aligned} R_{T}^g = \frac{1}{T-1}\sum _{t = 1}^{T-1} H\left( \Vert T^g_t - S^g_t \Vert - \Vert T^g_{t+1} - S^g_{t+1} \Vert \right) \end{aligned}$$

where *H* is the Heaviside step function.

The correct response rate is determined by the ratio of correct responses to given stimuli averaged over all players of the corresponding group. Although many times a correct response of a single player is accompanied and possibly partly overridden by actions of other players, an increase of the correct response rate over time indicates that individual players improve their capabilities leading to an increased group performance. We define the correct response rate for player $i \in \{1, \ldots , 4\}$ of group $g \in \{s, a\}$ up until time $T \le N$ to be

$$\begin{aligned} R_{T,i}^g = \frac{\sum _{t = 1}^T A_{t,i}^g H\left( \Vert T^g_t - S^g_t \Vert - \Vert T^g_t - \left( S^g_t + \phi \left( \mu _i^g\right) \right) \Vert \right) }{\sum _{t = 1}^T A_{t,i}^g} \end{aligned}$$

where *H* is the Heaviside step function.

#### Variance of spike triggering stimuli

In order to quantify the learning progress of individual players, we assume that the stimuli to which a single player reacts are distributed according to a von Mises distribution^93^ centered approximately around the player’s preferred direction. Over the course of game, players learn about the effect of their actions. Eventually, they react only to a subset of possible stimuli, which then results in a reduction of the variance of the fitted von Mises distribution over target difference vector angles. To calculate the variance, we transform the target difference vector at each point in time into a unit vector $x_t^g$ by

$$\begin{aligned} x_t^g = \frac{T^g_t - S^g_t}{\Vert T^g_t - S^g_t \Vert } \end{aligned}$$

We then approximate the circular variance ${\hat{\sigma }}_t^g$ at point *t* by

$$\begin{aligned} {\hat{\sigma }}_t^g = 1 - \left\Vert \frac{1}{t} \sum _{t' = 1}^t x_t^g\right\Vert , \end{aligned}$$

where the unit vector $\nu _x$ is the maximum likelihood estimation of the mean direction.^94^

#### Action time correlations

A tool to measure the group’s ability to work together is the action time correlation^95^ which can be thought of as a measure of similarity between two players’ response profiles. The idea behind this measure is that players with similar displacement direction should produce similar action sequences or spike trains over the course of a batch of trials.

The action time correlation between player *i* and *j* with $i,j \in \{1,\ldots ,4\}$ of group $g \in \{s,a\}$ for the data gathered in the Nested Neuron Game is defined as

$$\begin{aligned} C_{i,j} = \frac{\sum _{t = 1}^N (A^g_{t,i} - {\bar{A}}^g_i)(A^g_{t,j} - {\bar{A}}^g_j)}{(N-1)s_{A^g_i}s_{A^g_j}}, \end{aligned}$$

where $A^g_i$ and $A^g_j$ are binary column vectors of the matrix ${\textbf{A}}^g$, ${\bar{A}}^g_j$ is the sample mean across time and $s_{A^g_i}$ is the sample standard deviation.

#### Mutual information

In order to quantify the amount of information about the target position that is transmitted via the sensor group’s commands to the actor group, we determine the mutual information^96^ between the target position as seen by the sensor group and the action patterns of the actor group. The mutual information is calculated according to

$$\begin{aligned} I({\textbf{T}}^s, {\textbf{A}}^a) = -H({\textbf{T}}^s, {\textbf{A}}^a)+H({\textbf{T}}^s) + H({\textbf{A}}^a), \end{aligned}$$

where $H(\cdot )$ is a function that maps the matrices ${\textbf{T}}^s$ and ${\textbf{A}}^a$ onto the entropy of distribution of the respective samples.

The idea is that the ensemble of players functions as a group as soon as the sensor group is able to provide correct information about the target to the actor group. Hence, we expect the mutual information to grow over the time as the groups cooperate.

#### Cross correlation and maximizing lag

As the actor group’s actions depend on the signals sent by the sensor group, the lag that corresponds to the maximal cross correlation^97^ conveys information on how fast the actor group adapts and reacts to the target descriptions sent by the sensor group. Therefore, each player in the actor group should have its maximal cross correlation with sensor players at some positive but small lag and the sensor players their maximal cross correlation with actors at some negative lag.

We calculate the cross correlation with lag $\tau$ between the *spike trains* of two players across actor and sensor group as follows

$$\begin{aligned} {\hat{R}}_{A^g_i,A^{{\bar{g}}}_j}(\tau ) = \sum _{t = 1}^{N - \tau - 1} A^g_{t+|\tau |,i} \,A^{{\bar{g}}}_{t,j}, \end{aligned}$$

where $g, {\bar{g}} \in \{s,a\}$ with $g \ne {\bar{g}}$ are the group indicators and the maximizing lag *L* as

$$\begin{aligned} L_{A^g_i,A^{{\bar{g}}}_j} = {{\,\mathrm{arg\,max}\,}}_{\tau = 1, \dots , N} {\hat{R}}_{A^g_i,A^{{\bar{g}}}_j}(\tau ). \end{aligned}$$

The valid combinations of players, namely all pairs of sensor and actor combinations, are then displayed in matrix form.

### Computational models

**Fig. 7:**
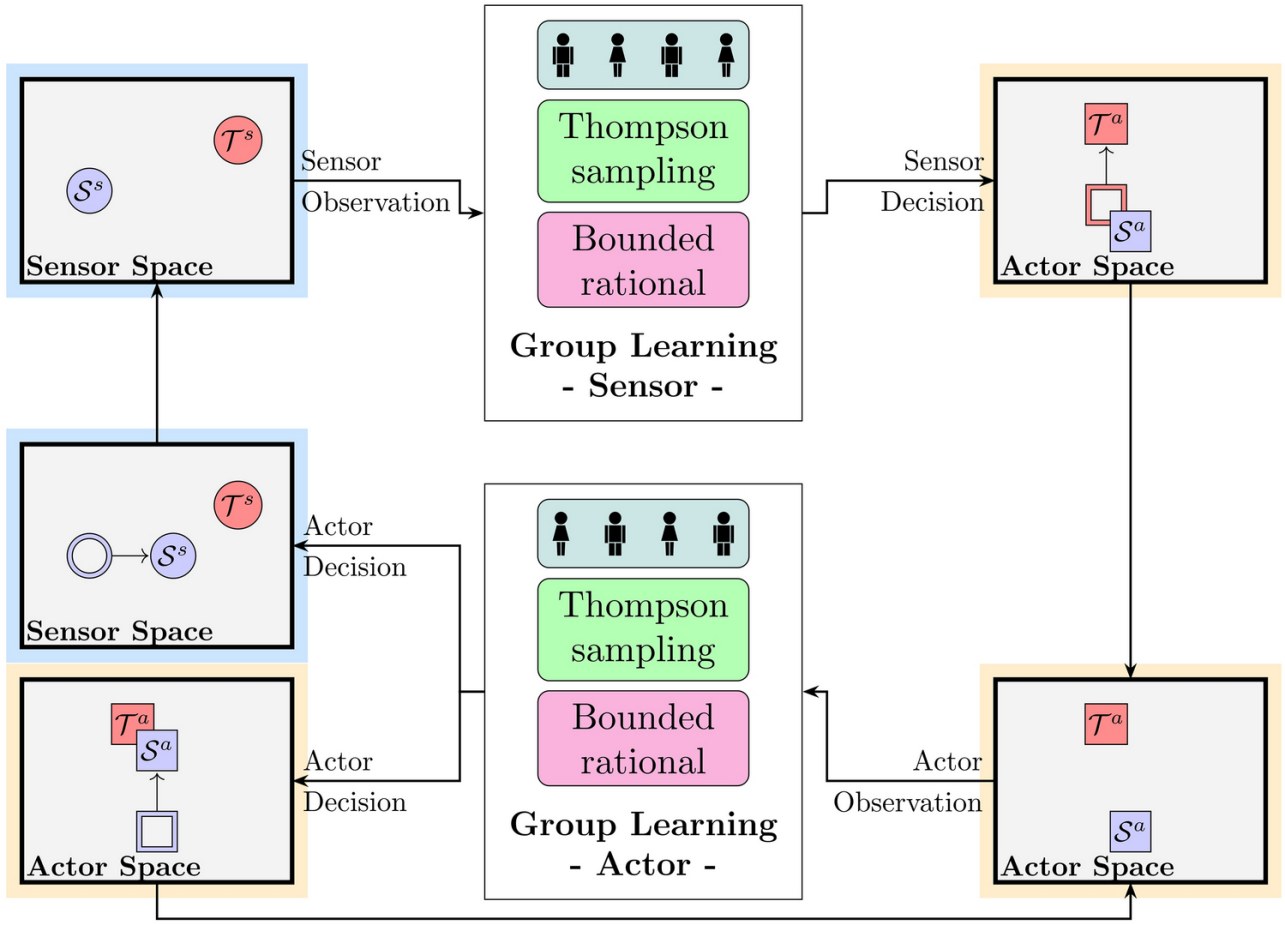
Overview over the key elements of the Nested Neuron Game. At every point in time the position of the cursors ${S}^s$ and ${S}^a$ together with the positions of the targets ${T}^s$ and ${T}^a$ incite a collective decision in both the sensor and the actor group. The sensor group decision causes a displacement of the target sprite on the actor screen generating an incentive for the actor players to adapt to the new target position. The actor decision causes a displacement of the cursor sprites on both the actor and the sensor screen leading to new observations for both groups. Over the course of the game each actor learns from their observations to react correctly to movement of their target, i.e. the input received from sensor players. Similarly, the sensor players learn which of their actions will cause specific reactions of their cursor sprite in the near future. To describe how the human participants learned to cooperate in the Nested Neuron Game we compare two different models that were also able to solve the Nested Neuron Game, a Thompson sampler that approximates Bayes optimal decisions and a system of bounded rational learners.

All models consist of four decision-making units that form the sensor group and four decision-making units that form the actor group. The groups are linked exactly like the participants that played the Nested Neuron Game, in that sensor players only receive the information on their screen, namely the coordinates of the cursor and the target, and actor players get only the coordinates of their respective cursor and target (see Fig. 7 for an overview of the key elements of the Nested Neuron Game).

To account for the human players’ sparse activity, the decision-making units are only allowed to make a decision at approximately $80\%$ of points in discrete time. This fraction was fitted to the data gathered in the experiment to ensure that the amount of players that are active at a specific point in discrete time is in distribution the same for simulated decision-makers and human participants (we assumed the amount of players that are active at each point in time is a binomially distributed random variable).

In contrast to the previous experimental setting in the Neuron Game,^16^ participants and simulated decision units in the Nested Neuron Game are split into two different groups with different learning tasks. While the actor group’s learning task is analogous to the learning task in the original Neuron Game experiment, in that every player has direct access to the consequences of the group’s combined action in the same time step, the sensor group’s decisions in the Nested Neuron Game cause observable consequences that are delayed by multiple time steps. In particular, the observation received by the sensor group consists of a mixture of delayed reactions of the actor group to a mixture of signals sent by the players in the sensor group.

**Fig. 8:**
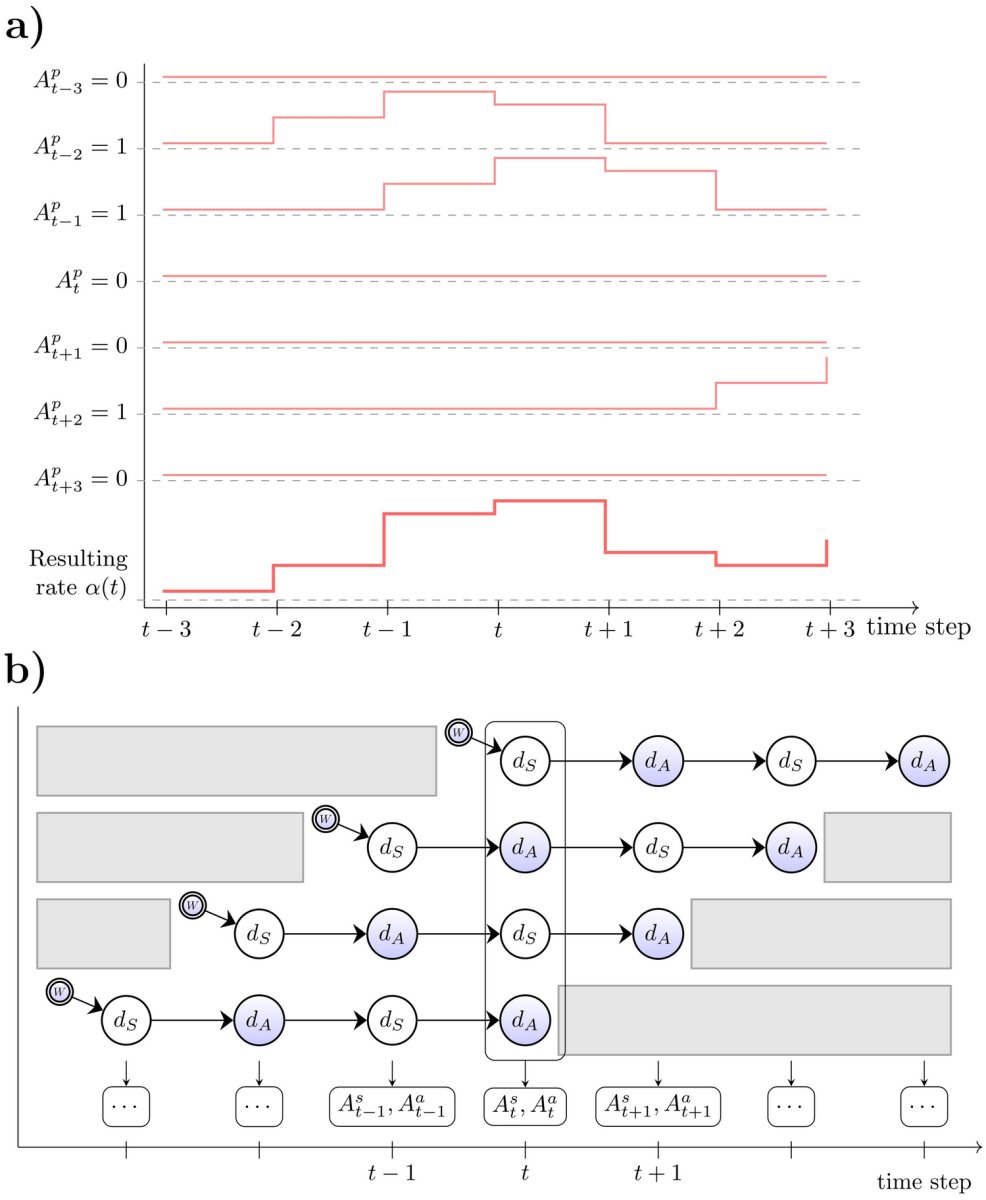
Time windows for learning. (**a**) Whenever units in the Bayes model are active the learning rate increase for the next few time steps. In the specific example, whenever the unit decides to be active, the learning weight for the time step and the following two time steps increases by 0.5, 1 and 0.75, respectively. The decisions $A^g _{t-2}$ and $A^g _{t-1}$ cause the maximum learning rate at time *t* with 1.75. (**b**) For the bounded rational decision-making model, at every time step the current worldstate is given to the network as an observation and a network sample is generated, such that each communication step in the network corresponds to a time step in the game. Actions are then randomly chosen from all actions that can occur at a specific time in the game. For example at time *t*, there are two possible decisions for the sensor group $d_S$ and two possible decisions from the actor group $d_A$. The action at time *t* is the drawn uniformly for the two decisions.

To tackle this problem in the following, we use a Bayesian learning model and a network of bounded rational decision-makers and we incorporate mechanisms that allow to track observations over a period of time and learn from it (see section Model details). In the Bayes model, this prolonged learning period is achieved by a time-dependent learning rate parameter $\alpha (t)$ that is adjusted such that after each action of a player, observations of the next time steps are incorporated into the existing Bayes estimate of the actual consequence of being active (see Fig. 8a for an example of the importance weight expressed by the learning rate $\alpha$ that is given to observations at specific points in discrete time). The action at each specific point in time of a sensor player then only depends on this estimate of the player’s preferred direction.

In the case of the network of bounded rational decision-makers we model the continued interaction between sensor an actor players using a loopy decision network, where the actions of sensor players influence the behavior of actor players, which in turn again changes the behavior of the sensor players and so forth. At each point in time the state of the sensor player world is then observed by the sensor players, resulting in a chain of alternating decisions from the sensor players and actor players, generating a rollout of network states for each point in time. Actions for the game are sampled uniformly from the different rollouts as illustrated in Fig. 8b.

### Model details

#### Model 1: Bayes model

The Bayes model consists of a Thompson sampler^51^ that approximates Bayes optimal decisions in unknown environments. To this end, each Bayesian decision-making unit maintains a record of all potential outcomes of their actions and assigns probabilities to indicate their level of plausibility. The possible outcomes in the game are possible displacement angles $\nu$ that occur after a player has been active. Analogously to the experiment, each decision-making unit has a binary action (or decision) space $\{0,1\}$ consisting of the decision 1, representing the action of pressing the button, and the decision 0, whenever the unit decides to stay idle. A Bayes-optimal agent chooses their actions so as to maximize the expected utility where the displacement $\nu$ is most likely to co-align with the target difference vector angle $\psi$. Thus, whenever a Bayesian decision unit observes a world state given by $\psi$, they become *active* if they believe this action is more beneficial than staying idle. This can be expressed by the utility function *U* given by

$$\begin{aligned} U(A,\psi , v) = {\left\{ \begin{array}{ll} 0 & \text {if } A = 0 \\ \frac{\pi }{2} - \measuredangle (\psi , v) & \text {if } A = 1 \end{array}\right. }, \end{aligned}$$

where $\measuredangle (\psi , v)$ is the angular distance between $\psi$ and *v*. Accordingly, the utility is zero, whenever the decision unit is inactive, and either positive or negative whenever the unit is active, depending on whether the angular distance is less or more than $90^\circ$, i.e. depending on whether the cursor is displaced in the right direction with respect to the target.

To determine the best action, we compare the expected utility between the two possible actions, i.e.

$$\begin{aligned} f = \langle U(A = 1, \psi , v) \rangle _p - \langle U(A = 0, \psi , v) \rangle _p = \langle U(A = 1, \psi , v) \rangle _p, \end{aligned}$$

where *p* is a belief over displacement directions $\nu$. The belief *p* over the movement directions can be expressed by a von Mises distribution $p_{\theta ^g_i}(v)$ over displacement angles *v* given by

$$\begin{aligned} p_{\theta ^g_i}(v) = \frac{1}{2\pi I_0(k)}e^{{\hat{\kappa }}_i^g \cos (v-{\hat{\mu }}^g_i)}, \end{aligned}$$

where $\theta ^g_i = ({\hat{\mu }}^g_i,{\hat{\kappa }}_i^g)$ denotes the mean and concentration of the distribution. The Thompson agent infers the two parameters by sampling the Bayes posterior of the parameters $\mu$ and $\kappa$:

$$\begin{aligned} p(\mu , \kappa \mid R,\Theta ,T) = \frac{e^{\kappa R \cos (\Theta -\mu )}}{K(R,T) I_0(\kappa )^T}, \end{aligned}$$

where $K(R,T) = 2\pi \int _0^\infty \frac{I_0(R\kappa )}{I_0(\kappa )^T} d\kappa$ is a normalizing constant and $I_0$ is the modified Bessel function of the first kind of order 0.^98^ Updates to the agent then consist of adapting the parameters *R* and $\Theta$, where given a (complex) resultant vector of observations $\{\psi _t\}_{t \in \{1,\ldots ,T\}}$ at time *T*

$$\begin{aligned} a + b\textrm{i} = \sum _{t = 1}^T \alpha (t)e^{\psi _t}, \end{aligned}$$

with time-dependent learning rate $\alpha (t)$ the parameters take the following form:

$$\begin{aligned} R&= |a+b\textrm{i}| \\ \Theta&= \text {atan2}(b,a) \mod 2\pi . \end{aligned}$$

Finally, the action probability is determined using a soft activation function $\sigma _\rho ^g(\cdot )$ on *f* and an action is sampled from this probability. The parameters $\rho$ are part of the hyper-parameters $\vartheta$. As an additional regulizer to prevent overfitting, we employ a preceding $\varepsilon$-greedy strategy, where an agents opts for either a random action with probability $\varepsilon$ or for an action sampled according to its action distribution with probability $1-\varepsilon$.

In summary, the set of parameters of the ensemble of eight units that are updated over the course of a batch of six trials is $(\theta _1^s,\ldots , \theta _4^s,\theta _1^a,\ldots , \theta _4^a)$ and the set of hyper-parameters that is shared across all members of a group is $(\vartheta ^s, \vartheta ^a)$. The hyper-parameters $\vartheta ^g_i$ are model specific and chosen in a way, such that the decision-units emulate the behavior of the participants. The parameters $\theta ^g_i$ are adapted over the course of the trials and reset after each batch of six trajectories.

In the simulation for the Nested Neuron Game, the inital parameters that were learned over the course of the trials were set to $R = 0$ and $\Theta = \text {NaN}$. The parameter of the sigmoid activation function, the parameter of the $\varepsilon$-greedy strategy and the time dependend activation function for all units were set to $\rho = 2.73$, $\varepsilon = 0.23$ and $\alpha (t) = 0.51 A_t + 1.03 A_{t-1} + 0.89 A_{t-2}$, where $A_t \in \{0,1\}$ is the action of the corresponding player at time *t*.

#### Model 2: bounded rational decision network

The second model consists of a loopy, two-layer network composed of two sets of four bounded rational decision-makers,^54^ each trying to optimize a local utility function. These decision-makers are connected according to the information flow dictated by the dynamics of the Nested Neuron Game. At each point in time, each decision-maker $s_i$ in the first layer, i.e., the sensor layer, receives information about the target difference vector by observing the output of the world-state node *w*. Additionally, they have access to the actions of all nodes $a_j$ in the actor layer. The sensors $s_i$ then process the information obtained and choose an action (or decision) from the binary action space $\{0,1\}$, where the decision 1 represents pressing the button, and the decision 0 represents remaining idle.

Similarly, each actor $a_j$ observes the output of all sensors $s_i$, processes the information, and acts accordingly. Here, processing information means that, given an input, the decision maker transforms its prior probability distribution over actions into a posterior distribution, and choosing an action consists of sampling an action from said posterior distribution.

$$\begin{aligned} p^t(X_i^g)&\rightarrow P^t(X_i^g \mid \cdot )\, \text { and}\\ A_i^g&\sim P^t(X_i^g \mid \cdot ) \end{aligned}$$

Unlike perfect rational decision-makers that always choose the perfect action to maximize a given utility function, the behavior in the bounded rational network agents is assumed to be stochastic, in that they cannot always determine the best action, but simply choose an action that is good enough on average, as they balance the cost of information processing with a gain in utility.

This trade-off can be expressed in terms of a product between prior and utility for each decision unit, i.e.

$$\begin{aligned} P^t(X^s_i \mid W,X^a_{1,2,3,4})&\propto p(X_i^s) e^{\beta ^s_i{{\hat{U}}^{s}_i}(X^s_i,W,X^a_{1,2,3,4}; t)} \, \text { and} \\ P^t(X^a_i \mid X^s_{1,2,3,4})&\propto p(X_i^a) e^{\beta ^s_i{{\hat{U}}^{a}_i}(X^a_i,X^s_{1,2,3,4};t)}, \end{aligned}$$

where *P* and *p* denote the posterior and prior, respectively, $\beta$ is a (hyper-) parameter that interpolates between a rational agent ($\beta \rightarrow \infty$) and an agent that behaves randomly $(\beta \rightarrow 0$), and where ${\hat{U}}$ is the effective utility of the agent at a specific point in the decision-making process. The effective utility combines each layer’s utility for getting closer to the target with the information processing costs arising from subsequent decision-makers in the decision-making process.^54^ For the simulations in the Nested Neuron Game we chose a depth of the decision-making process of 4 and temperature parameters of $\beta = 2.3$.

**Fig. 9:**
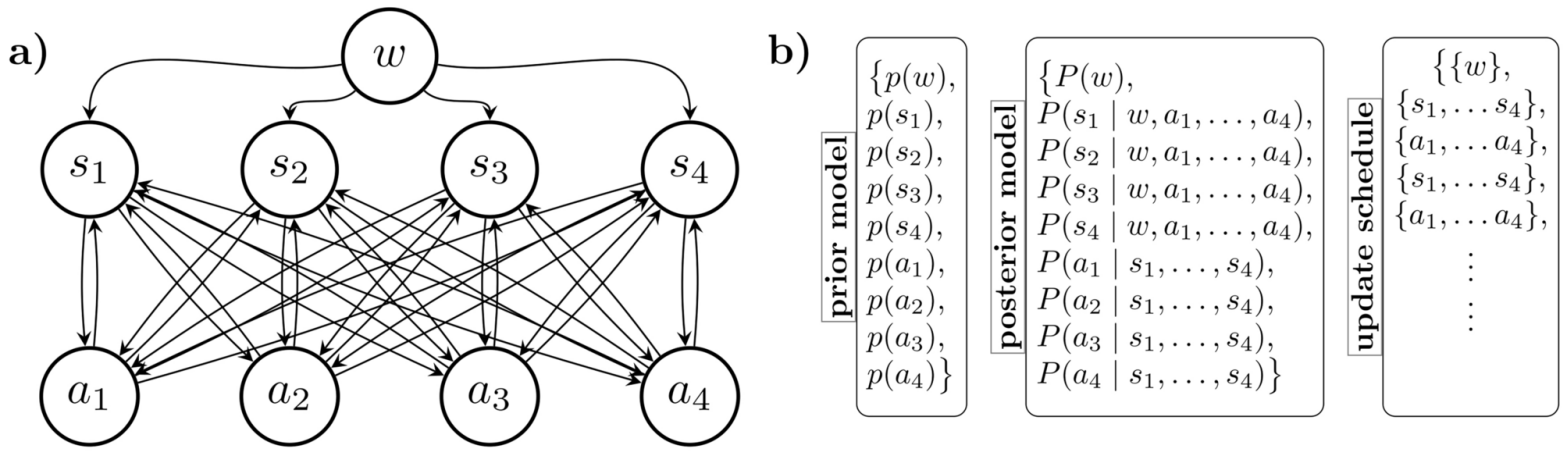
Bounded rational decision network. (**a**) Graphical model representing prior and posterior models. (**b**) Communication structure consisting of prior and posterior model, and update schedule.

Due to the Nested Neuron Games dynamics, information is flowing back and forth from sensors to actors and vice versa, creating a feedback loop, where actors react to sensor signals, that in turn were responses to previous actor actions and so forth. The resulting highly connected, loopy decision-network (see Fig. 9a) displays the prior and posterior models of the decision-making process, i.e. it describes with whom each node communicates (see Fig. 9b). As there is no linear order of nodes in a loopy network, there is no sequence of information processing steps of the involved units that can be derived from the architecture of the decision network. Therefore, we define an update schedule in which sensors and actors alternately update their probability distributions (see Fig. 9b), describing when the involved units communicate.

The Nested Neuron Game is then simulated as follows: At each point in time, given a discretized version of the target difference vector angle ${\hat{\psi }}$, the decision network computes a rollout of actions of all involved decision-makers by sampling from the corresponding posterior distributions, generating action sequences for the agents in the sensor layer as well as for the agents, that are translated into trajectories of the sensor and actor cursor as well as the actor target. As agents start the game by acting randomly and gradually become more certain of their actual contributions to the overall movement, we initialize all decision-making agents with $\beta$ parameters close to zero, increasing them over time.

#### Hyperparameter tuning

In order to find sets of parameters (see section Model details) for which the models’ performance was close to human performance, we conducted a two-step hyperparameter tuning process.

In the first step, we performed a broad grid search over the parameter space to identify configurations that allowed the simulations to successfully complete the task. Once successful parameter regions were identified, we conducted a second, finer grid search. This step focused on refining the parameter values to maximize the similarity between the simulated results and human performance. Key metrics for comparison included the length of cursor trajectories, improvement rates, and mutual information between the sensor target and the vector of actor actions.

This two-step approach ensured that the simulations were not only capable of completing the task but also aligned closely with the behavioral patterns observed in human participants.

### Evolutionary algorithm

We performed simulations and modified the Nested Neuron Game to allow for arbitrary network structures, by varying the number of layers as well as the number of units per layer. To describe the connections between the layers, we dub the sensor layer $L_0$ and all other (actor) layers $L_i$ with $i \in \{1,2,\ldots \}$. The network is defined by listing the layers together with the corresponding decision agents and two weight matrices, $M_{\mathcal {S}}$ and $M_{\mathcal {T}}$, with entries $w_{k,l}$ that describe the magnitude of displacements caused by actions in layer *k* that are observed in layer *l*.

To optimize the network architecture, we use an evolutionary approach,^56,57^ where starting from an initial network, the number of layers, the number of agents per layer, the weight matrices $M^{\mathcal {S}}$ and $M^{\mathcal {T}}$, and the $\varepsilon$ parameter of an $\epsilon$-greedy strategy employed by the units are allowed to mutate. We chose the Thompson sampling model as a base model for each decision-maker of the evolving networks and kept all other hyper-parameters of the learners fixed. Additionally, we investigated three different sets of restrictions on the mutations allowed by the evolutionary process (see Fig. 6a–c for an illustration):

- Scenario 1: $M^{\mathcal {T}}_{j,1} = 0$ for all *j* and $M^{\mathcal {S}}_{1,1} = 0$, i.e. no layer can move the sensor layer’s target (it is given externally) and the sensor layer agents cannot change their own position.
- Scenario 2: $M^{\mathcal {T}}_{j,1} = 0$ and $M^{\mathcal {T}}_{j,j} = 0$ for all *j* and $M^{\mathcal {S}}_{1,1} = 0$, i.e. the same restrictions as before, and additionally, no agent is allowed to move the target of its own layer.
- Scenario 3: $M^{\mathcal {T}}_{j,1} = 0$ and $M^{\mathcal {T}}_{j,j} = 0$ for all *j* and $M^{\mathcal {S}}_{1,1} = 0$, i.e. the same restrictions as before and additionally, the network is forced to work strictly sequentially (like a feedforward network), i.e. $M^{\mathcal {T}}_{k,l} = 0$ for all entries that do not lie on the upper secondary diagonal and $M^{\mathcal {S}}_{k,l} = 0$ for all entries that lie neither on the diagonal nor the lower secondary diagonal.

The main objective for the evolutionary optimization was maximizing the performance of the network structure, i.e. minimizing the number of steps required to solve ten instances of the Neuronal Neuron Game. Thereby, the population-based metaheuristic optimization had to find a balance between increasing and decreasing the number of involved agents and the $\epsilon$ parameter.

In the Number of units and layers within the ensemble section of the Results we utilized a population-based meta-heuristic algorithm to optimize the performance of the systems that played the modified version of the Nested Neuron Game that did not have any restrictions on the number of layers of the systems or the number of units within each of the layers. To this end, we used handcrafted systems with two, three, four and five layers with eight to 18 units in total that were able to solve the game as initial inputs for the evolutionary process. Then, the initial system was mutated 99 times, forming the first generation of 100 systems that played five repetitions of the Nested Neuron Game with 12 trials each. For each system the performance was measured as the number of steps it required to complete the game 5 times. In each generation the top 30% of systems survived and were kept for the next generation. Additionally, the top 10% of systems had three, all other surviving units had two (randomly mutated) descendants. Mutations included:

- Increasing or decreasing the number of layers by one, down to a minimum of two layers.
- Increasing or decreasing the number of units in every layer by one, down to a minimum of three units.
- Increasing or decreasing the value describing the connection strength between layers for both, displacements of the target (or reference value) as well as disturbances of the positions (or state). All values contained in the matrices were limited to the interval between $-1$ and 1.
- Increasing or decreasing the number of steps the units record to learn about their preferred direction, down to a minimum of 1 step.
- Increasing or decreasing the $\epsilon$ parameter of an $\epsilon$-greedy strategy employed by the units. The parameter choice was limited to the interval [0, 0.5].

## Supplementary Information

Supplementary Information.

## Data Availability

The datasets collected during the study are available from the corresponding author upon request and will later be made publicly available in the OPARU open access repository of Ulm University.
