# Supplementary material for: Human intergroup coordination in a hierarchical multi-agent sensorimotor task arises from concurrent co-optimization
Source: Sci Rep. 2025 Apr 28;15:14849. doi: 10.1038/s41598-025-97574-3 (PMC12038055; doi:10.1038/s41598-025-97574-3)
Supplement: Supplementary file 1 — Supplementary Information. [file 41598_2025_97574_MOESM1_ESM.pdf]

Human intergroup coordination in a hierarchical multi-agent sensorimotor task arises from concurrent co-optimization

Gerrit Schmid[1,*] **and Daniel A. Braun**[1]

[1]Faculty of Engineering, Computer Science and Psychology, Institute of Neural Information Processing, Ulm University, 89081 Ulm, Germany
[*]gerrit.schmid@uni-ulm.de

Supplementary material:

Intergenerational evolution of performance and network architecture

We provide additional visualization of the results of the evolutionary algorithm that is used to optimize the performance of the networks in the Nested Neuron Game that were described in the subsection Number of units and layers within the ensemble of the results part of the study. The algorithm iteratively mutated the network's parameters, among others the number of units and layers in the network, the level of stochastic behavior within each unit, and the connection strength between layers of units, which dictates the extent of their mutual influence. The figures S1, S2 and S3 offer a more granular view of the evolution across generations, providing further context to the trends discussed in the main text. Our results indicate that networks with higher stochasticity tend to learn faster, while an increased number of units correlates with improved overall performance.

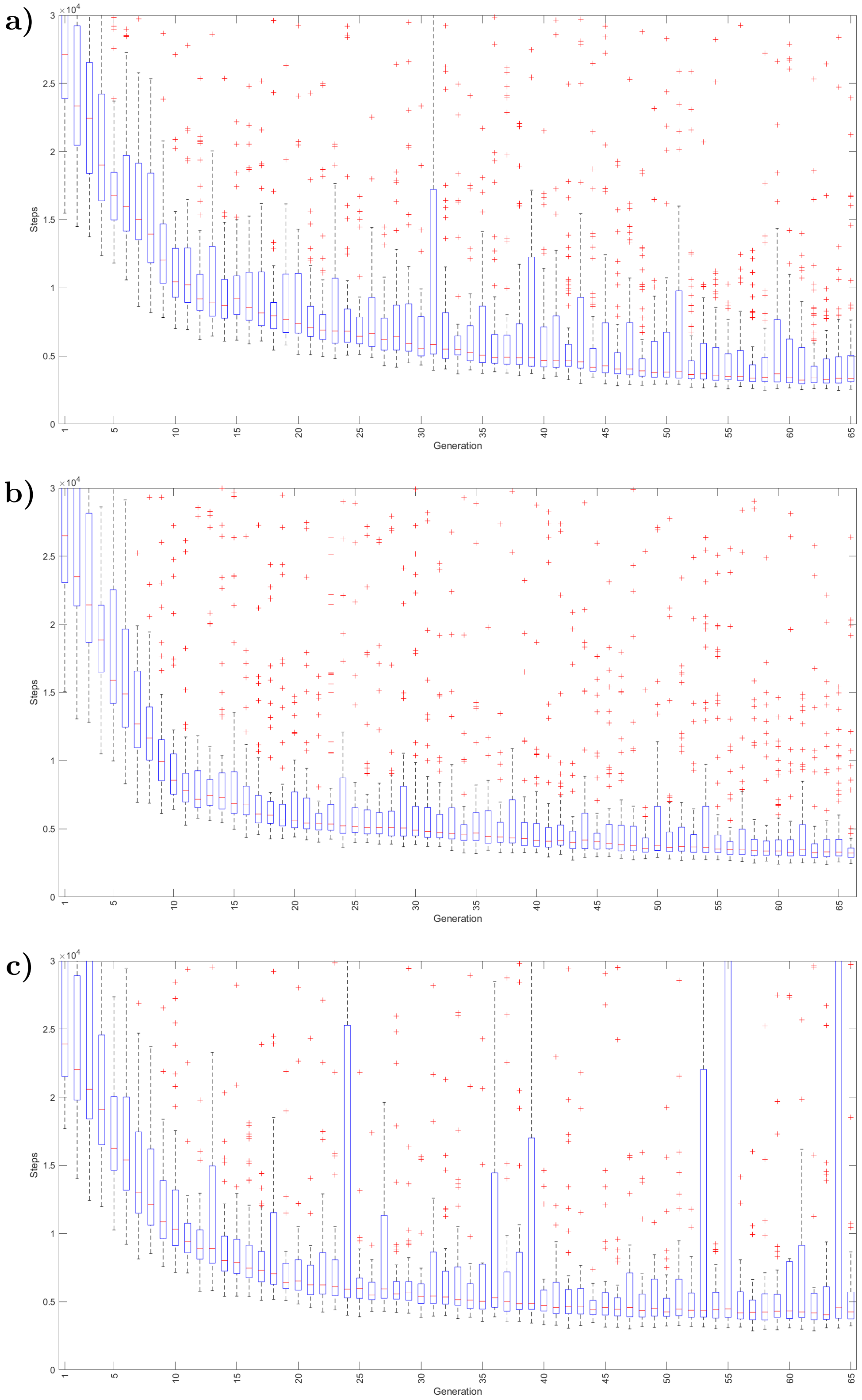

Figure S1. Steps required per generation. Boxplots showing the performance of an exemplary evolution of a multilayer extension of the learner introduced in the main text. Each Generation consists of 100 different learning systems. **a)** Performance of type 1 evolution, where layers $L_{>0}$ can influence their own target value (reference signal) and the target value of other layers $L_{>0}$. **b)** Performance of type 2 evolution, where each layer can influence only the target values of other layers $L_{>0}$. **c)** Performance of type 3 evolution, where each layer L_i can influence only the target values of the next layer L_{i+1}.

a)

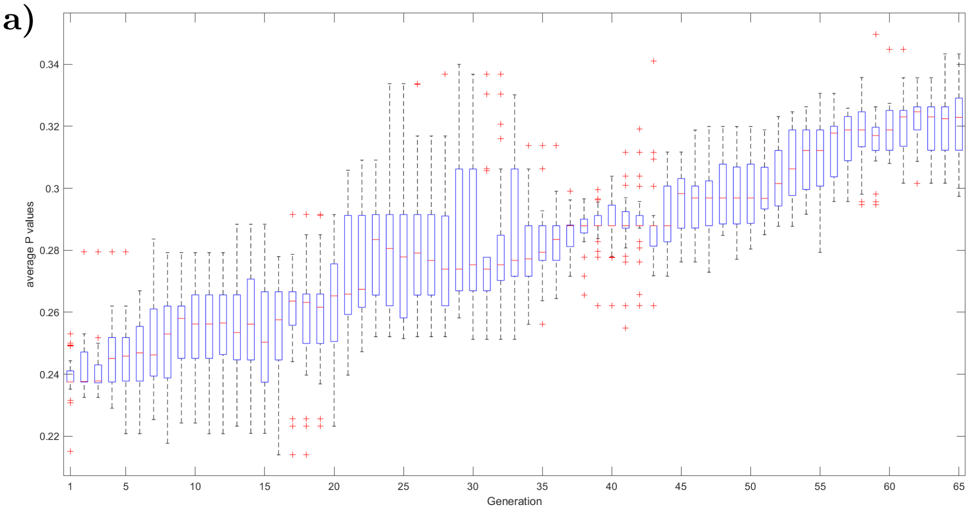

b)

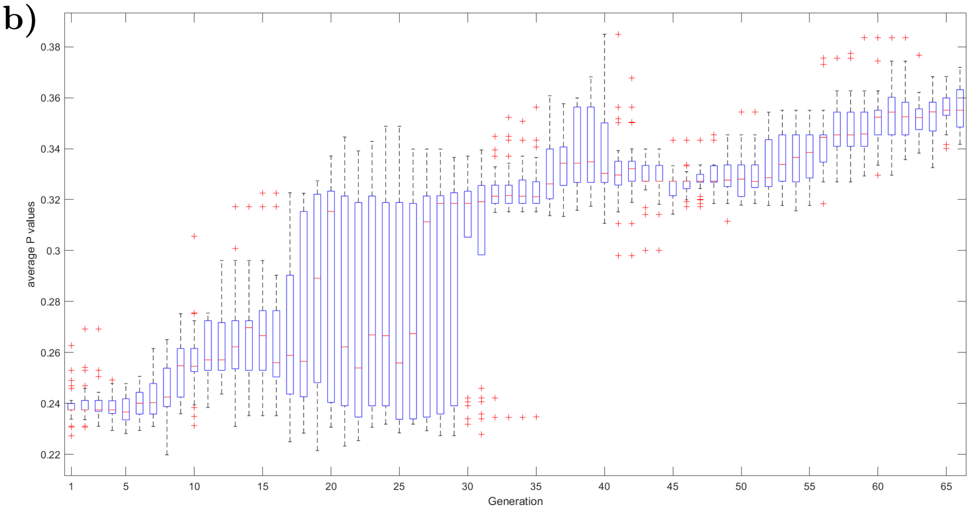

c)

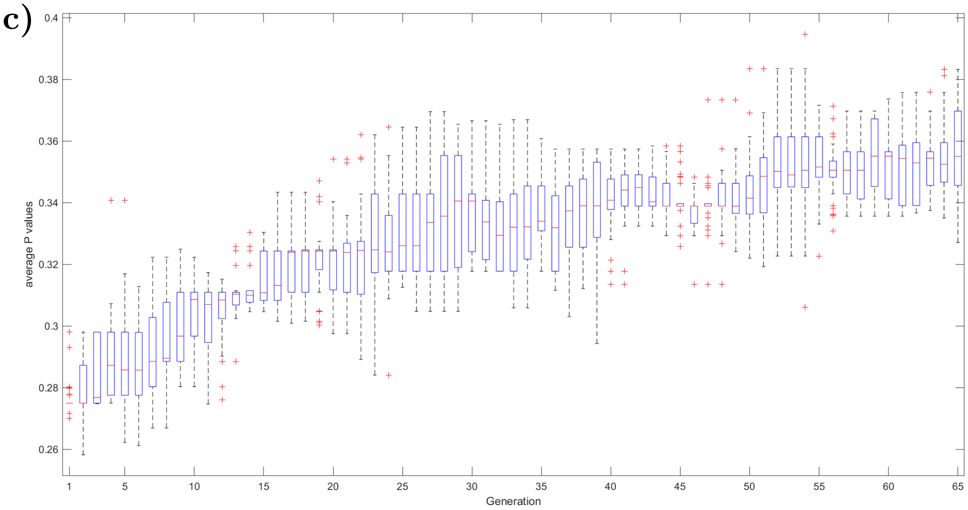

Figure S2. Frequency of random actions. Boxplots showing the frequency of random actions of decision-units in the top 30 systems of a generation of an exemplary evolution of a multilayer extension of the learner in the main text. Each Generation consists of 100 different learning systems. **a)** Frequency of random actions of type 1 evolution, where layers $L_{>0}$ can influence their own target value (reference signal) and the target value of other layers $L_{>0}$. **b)** Frequency of random actions of type 2 evolution, where each layer can influence only the target values of other layers $L_{>0}$. **c)** Frequency of random actions of type 3 evolution, where each layer L_i can influence only the target values of the next layer L_{i+1}.

a)

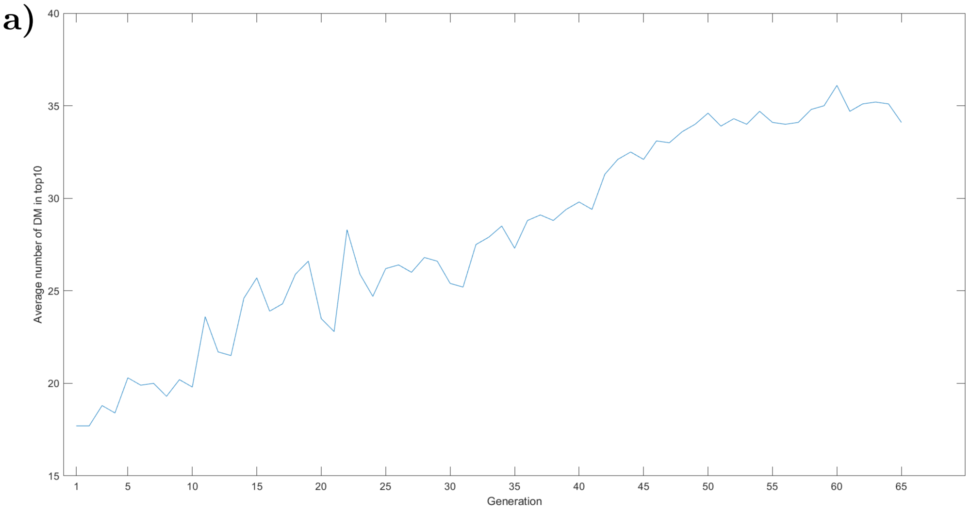

b)

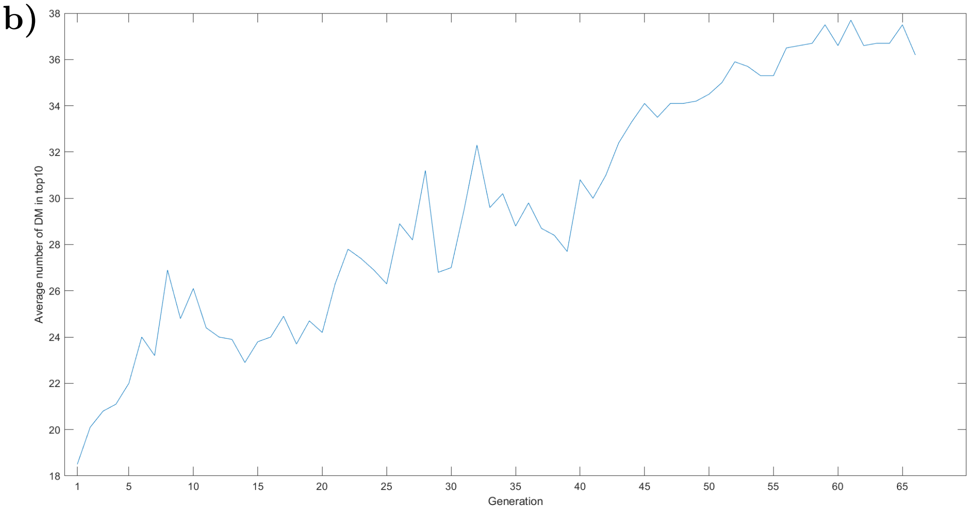

c)

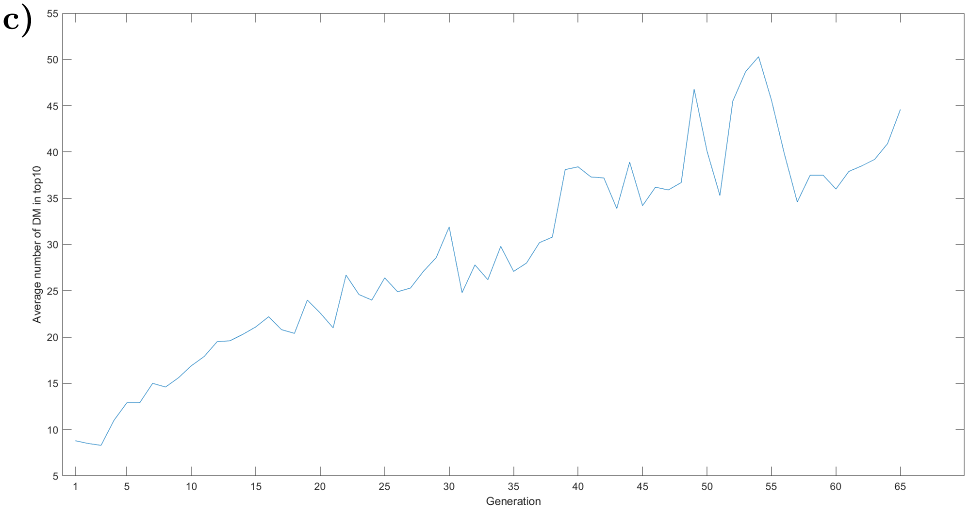

Figure S3. Frequency of random actions. Boxplots showing the number of decision-units in the top 10 generations of an exemplary evolution of a multilayer extension of the learner in the main text. Each Generation consists of 100 different learning systems. **a)** Number of units within systems per generation in a type 1 evolution, where layers $L_{>0}$ can influence their own target value (reference signal) and the target value of other layers $L_{>0}$. **b)** Number of units within systems in a type 2 evolution, where each layer can influence only the target values of other layers $L_{>0}$. **c)** Number of units within systems in a type 3 evolution, where each layer L_i can influence only the target values of the next layer L_{i+1}.
